# Control of division in *Chlamydomonas* by cyclin B/CDKB1 and the anaphase-promoting complex

**DOI:** 10.1371/journal.pgen.1009997

**Published:** 2022-08-18

**Authors:** Kresti Pecani, Kristi Lieberman, Natsumi Tajima-Shirasaki, Masayuki Onishi, Frederick R. Cross

**Affiliations:** 1 The Rockefeller University, New York, New York, United States of America; 2 Department of Biology, Duke University, Durham, North Carolina, United States of America; Peking University, CHINA

## Abstract

In yeast and animals, cyclin B binds and activates the cyclin-dependent kinase (‘CDK’) CDK1 to drive entry into mitosis. We show that CYCB1, the sole cyclin B in *Chlamydomonas*, activates the plant-specific CDKB1 rather than the CDK1 ortholog CDKA1, confirming and extending previous results. Time-lapse microscopy shows that CYCB1 is synthesized before each division in the multiple fission cycle, then is rapidly degraded 3–5 minutes before division occurs. CYCB1 degradation is dependent on the anaphase-promoting complex (APC). Like CYCB1, CDKB1 is not synthesized until late G1; however, CDKB1 is not degraded with each division within the multiple fission cycle, but is degraded after all divisions have ceased. The microtubule plus-end-binding protein EB1 labeled with mNeonGreen allowed detection of mitotic events in live cells. The earliest detectable step in mitosis, splitting of polar EB1 signal into two foci, likely associated with future spindle poles, was dependent on CYCB1. CYCB1-GFP localized close to these foci immediately before spindle formation. Spindle breakdown, cleavage furrow formation and accumulation of EB1 in the furrow were dependent on the APC. In interphase, rapidly growing microtubules are marked by ‘comets’ of EB1; comets are absent in the absence of APC function. Thus CYCB1/CDKB1 and the APC modulate microtubule function and assembly while regulating mitotic progression. Genetic results suggest an independent additional role for the APC in regulating sister chromatid cohesion; this role is likely conserved across eukaryotes.

## Author summary

We analyze progression and regulation of the cell division cycle in *Chlamydomonas reinhardtii*, a microbial member of the plant kingdom, compared to the well-studied yeast and animal systems. We analyze a battery of mutants defective in cell cycle progression by single-cell time-lapse microscopy of wild type and mutants. We find striking conservation of many aspects of cell cycle control, including the roles of the anaphase-promoting complex (APC) and the proximal role of cyclin B as a mitotic inducer; surprisingly, cyclin B in *Chlamydomonas* activates a plant-specific cyclin dependent kinase CDKB, rather than CDK1 as is the case in yeast and animals. Changes in microtubule structures are closely correlated and causally dependent on changes in cyclin B and APC activity.

## Introduction

Progression through the cell cycle in eukaryotes is controlled by cyclins and cyclin-dependent kinases (CDKs) [1]. CDKs generally lack any protein kinase activity as monomers; formation of a cyclin-CDK heterodimer causes a major refolding of the CDK to a kinase-active form [2]. The cyclin-CDK interface is large, and binding is essentially irreversible. In addition to activation of CDK enzymatic activity, cyclins frequently also contribute ‘docking sites’ that can bind to substrates, thus providing much of the substrate specificity for the heterodimer [3]. In most eukaryotes there are multiple CDKs and cyclins, which may differ in abundance and specificity toward particular targets at each cell cycle stage. In budding yeast, for example, G1 phase functions and initiation of the cell cycle are driven by the G1 cyclins, Cln1-3, and mitosis is driven by the B-type cyclins, Clb1-4 [4]. B-type cyclins are required for entry into mitosis in every eukaryote tested. In many systems, cyclin B degradation then becomes essential for mitotic exit (telophase and cytokinesis) [5,6]. Independently, relicensing of replication origins is also blocked by high CDK activity in many organisms [7].

The dependence of CDKs on cyclins for enzymatic activity and frequently for substrate targeting means that CDK activity can be readily controlled indirectly, by control of cyclin abundance, and both transcriptional and post-transcriptional control of cyclin levels are major cell cycle control mechanisms. Cyclin degradation is necessary to eliminate the complex, since cyclin-CDK binding is essentially irreversible. Cyclin B degradation occurs by proteolysis after ubiquitination by the anaphase-promoting complex (APC), a multi-subunit E3 ubiquitin ligase [8]. The APC is activated by Cdc20 and Cdh1 [9,10]. Ubiquitination is frequently dependent on a destruction box (‘D box’) consensus sequence on the target cyclin [11]. In budding yeast, genomic removal of the destruction box sequence from various B-type cyclins has been shown to stabilize their levels [12,13]. In contrast to cyclins, CDK1 in most systems is thought to be highly stable and in excess throughout the cell cycle, at least in rapidly propagating cells [1].

The APC is essential in almost all eukaryotes; one remarkable exception is *Giardia*, which lacks genes for all APC subunits [14]. *Giardia* cyclin B lacks a destruction box, but an unknown system nevertheless degrades cyclin B in mitosis. In most eukaryotes, the APC is required for anaphase independently of its role in cyclin B degradation, since it is required to remove securins, which are inhibitors of sister chromatid separation and anaphase [10]. Securin blocks activation of the ESP1 (separase) protease. Separase is required for proteolysis of the cohesin complex, releasing sister-chromatid cohesion and allowing anaphase. In yeast, a separate role for ESP1 was proposed in promoting mitotic exit [15], including regulation of mitotic spindle stability [16]. It is unclear if these separate roles are specific to yeast; separase inactivation did not block the DNA replication cycle in mouse cells [17]. In animal cells, separase may also promote centriole disengagement and duplication [18].

In budding yeast, while degradation of the B-type cyclin Clb2 is essential for mitotic exit [13], the closely related B-type cyclin Clb3 can persist undegraded without blocking mitotic exit or affecting viability [12]. In addition, the requirement for APC-dependent cyclin B proteolysis can be bypassed by periodic inhibition of cyclin B-CDK-associated kinase [19]. In *Drosophila* embryogenesis, there is little or no cyclin B degradation through the first 8 cycles, and mitotic exit is likely controlled instead by periodic inhibitory CDK phosphorylation [20]. Thus, cyclin B degradation is observed and essential for mitotic exit in many but not all eukaryotic cell divisions.

The plant kingdom, including the green alga *Chlamydomonas*, diverged from yeast and animals early in evolution [21]. This early divergence means that it is unsafe to assume conservation of regulatory systems without direct evidence; even highly homologous proteins can play different roles in plants compared to yeast and animals [22,23]. *Arabidopsis thaliana* contains multiple cyclins clustered into groups with specific functions depending on the cell cycle stage [24–26]. In yeast and animals, the CDK activated by cyclin B to drive mitosis is CDK1 [1]. All members of the plant kingdom contain a CDK1 ortholog called CDKA. Surprisingly, the plant-specific CDKB1, which is significantly diverged from CDK1, is the essential CDK for mitotic entry, rather than CDKA1 [27,28], and CDKB1-associated kinase activity was genetically dependent on CYCB1 [29]. CDKA1 instead is largely restricted to a role at cell cycle initiation [27,30,31].

Overall, these data suggest that early in the evolution of the plant lineage, the plant-specific CDKB1 took over the role of inducing mitotic progression in response to cyclin B accumulation. Experiments in another unicellular green alga *Ostreococcus* support this idea [32]. It is also noteworthy that clear CDKB1 orthologs are found in early-divergent algae of Glaucophyta and Rhodophyta [33,34], and although their partner cyclins have not been determined, a glaucophyte *CDKB1* transcript is strongly induced during cell division [33]. Moreover, “divergent” *CDKA2* genes are found in many heterokont algae (who share ancestry with plants only near the root of the eukaryotic tree), some of which may be induced during cell division [23,35]. Thus, CDK fluctuation and/or divergent CDKB (-like) genes may have originated very early during the evolution of the Archaeplastida lineage.

As in yeast and animals, some plant cyclins are controlled by destruction box- and proteasome-dependent degradation [36], suggesting that APC plays an important role in regulating CDK in plants. In addition, the activity of CDKB1 may also be regulated directly through its own degradation [32,37], suggesting an additional plant-specific layer of regulation. In *N*. *tabacum*, expression of destruction-box-deleted *CYCB1* resulted in defective cytokinesis [38], perhaps due to inactivation of cytokinesis-inducing proteins by CDK phosphorylation [39]. Thus, CDK/cyclin regulation in plants may diverge from the canonical models developed in animals and yeast, and elucidation of such regulation is likely to allow us to dissect evolutionarily conserved fundamental core mechanisms as opposed to ancillary modules that evolved in independent lineages. However, several aspects of plant biology pose challenges to genetic analysis in land plants [40].

The green alga *Chlamydomonas reinhardtii* offers considerable technical advantage to study CDK/cyclin regulation (and many other processes) in the plant kingdom. Its phylogenetic positioning near the base of Viridiplantae means that its ‘plant-like’ genome has not gone through multiple genome duplication events, which, together with the unicellular lifestyle and haploid genome, significantly facilitates genetic and cell biological studies of cell division cycle [40].

*Chlamydomonas* exhibits a pattern of cell division called ‘multiple fission’ [40]. Small newborn cells can grow over a 10–12 hr period to >10-fold their starting size without DNA synthesis or cell division. Cells then resorb cilia and undergo multiple rapid cell divisions: complete rounds of DNA replication, nuclear division, and cytokinesis, all within the mother cell wall, until progeny cells have divided to approximately their starting size [40]. Each cycle requires ~30 min, and all the newborn cells are retained within the mother cell wall until hatching occurs after the terminal cell division [40]. Transcriptome analysis of diurnally synchronized populations showed a large cluster of genes that are strongly induced throughout the multiple cycles of S-M-C [41–44]. This cluster includes *CDKB1* and *CYCB1*; the timing of induction of this cluster was strongly delayed by removal of CDKA1 [30,43], consistent with the idea that CDKA1/CYCA1 is largely responsible for cell-cycle initiation, while CDKB1/CYCB1 controls events involved in each S-M-C cycle.

Previously, we isolated ts-lethal mutations in multiple APC subunits, as well as in cyclin B and CDKB [28,29,45]. APC inactivation in *Chlamydomonas* results in arrest after one round of DNA replication with a metaphase spindle, suggesting that *Chlamydomonas* APC may be directly required for anaphase. This is likely true in land plants as well [46–49], probably due to a requirement for APC-dependent degradation of the Patronus anaphase inhibitor, a securin homolog [50]. APC-deficient plants also show delays in cyclin B degradation. In *Chlamydomonas*, mutational inactivation of the APC prevented down-regulation of CDKB1-associated protein kinase activity [29].

Microtubules play essential and diverse roles in mitosis, and cyclin B-dependent kinases may regulate dynamics and formation of mitotic microtubule-based structures [51,52]. Microtubules in *Chlamydomonas* include very stable ‘rootlets’ forming a cruciate structure centered on the basal bodies, containing acetylated tubulin [53,54]. In addition, there are unacetylated and highly dynamic microtubules extending from the vicinity of the basal bodies and rootlets and forming a cup-shaped pattern with the basal bodies as the base [53,55]. In mitosis, completely distinct microtubule structures form: the mitotic spindle and microtubules marking the cytokinetic furrow, which are likely essential for chromosome segregation and cytokinesis, respectively [56,57].

The microtubule end-binding protein 1 (EB1) binds to the plus end of actively polymerizing microtubules [58–60]. In yeast and animal cells, EB1 and its homologs are associated with cortical microtubules in interphase, appearing as small ‘comets,’ and associated with spindle microtubules in mitotic cells [61–65]. EB1 shows a similar localization pattern in *Arabidopsis*, where it appears as cortical comets during interphase [66], and is in the vicinity of spindle poles during mitosis [67]. In interphase *Chlamydomonas* cells, EB1 labeled with mNeonGreen (EB1-NG) is located in one or two anterior spots at or near to the ciliary basal bodies [55,57], and moving EB1-NG comets extend along the cell cortex to the cell posterior [55,57]. Moreover, in dividing cells, EB1-NG colocalizes with the spindle and the cleavage furrow [57], making it a useful marker to monitor mitotic events.

Here, we developed long-term quantitative single-cell imaging methods to examine kinetics of cyclin B and CDKB accumulation and degradation through complete multiple fission cycles and at mutant arrests in single cells, and correlate this behavior with the cytology of mitotic progression using fluorescently tagged EB1 as a reporter of microtubule localization, especially of the rapidly growing microtubule plus ends.

## Results

### CYCB1 interacts with CDKB1

B-type cyclins are key regulators of the cell cycle in animals and in yeast [1]. *Chlamydomonas* has a single essential cyclin B gene, *CYCB1* [29]. We constructed a transgene fusion with GFP appended to the C-terminus of CYCB1, under control of the *CYCB1* promoter, and identified transgene transformants that rescued lethality of a temperature-sensitive (ts) *cycb1-5* (Methods). We chose a transformant with a single GFP-containing locus that efficiently rescued *cycb1-5* in tetrad analysis. Following current *Chlamydomonas* naming conventions (https://www.chlamycollection.org/content/uploads/2021/11/Nomenclature-Final.pdf), we refer to this locus as *CYCB1*:*GFP-TG* (trans-gene).

**Fig 1:**
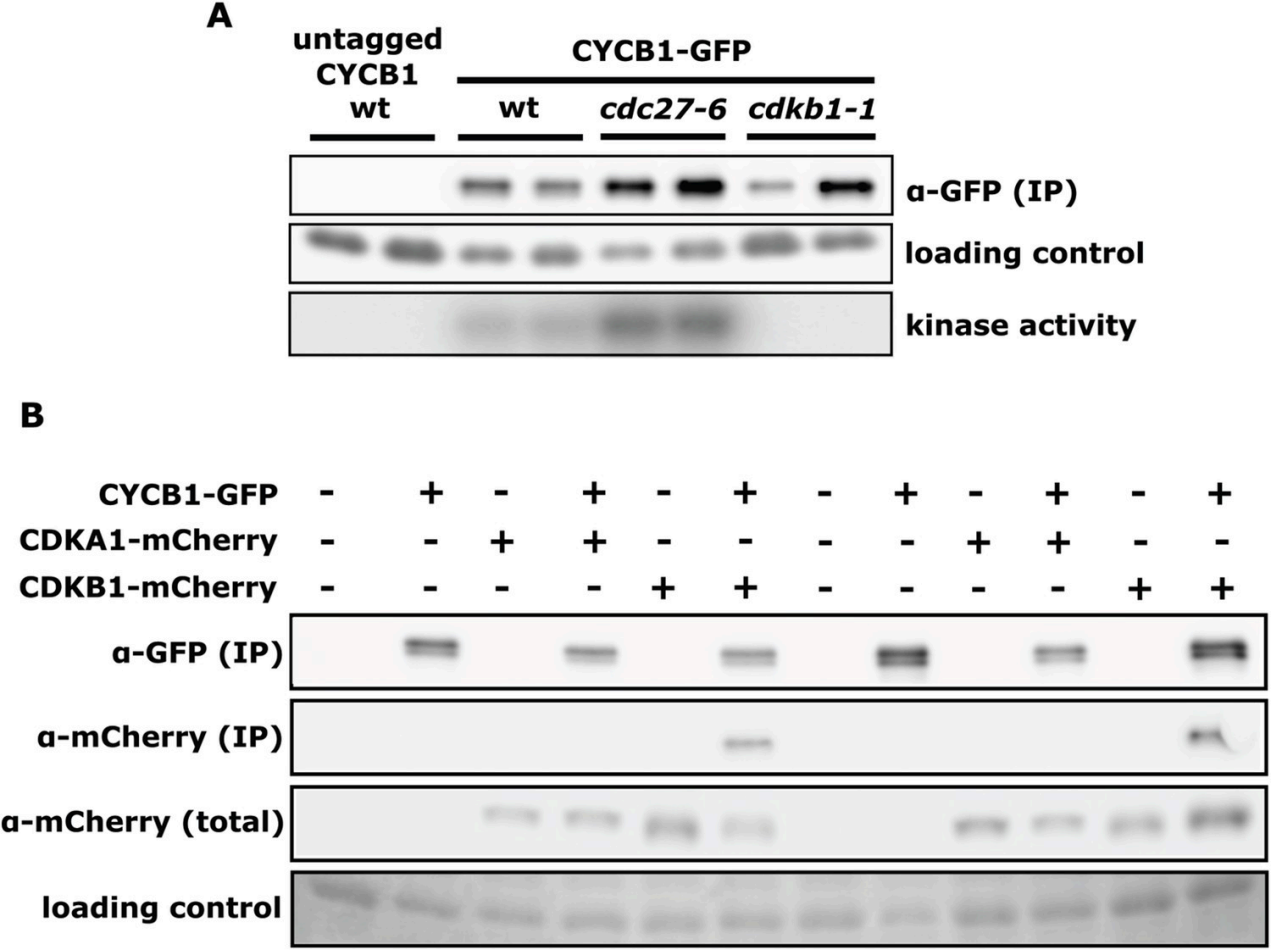
Detection of CYCB1-GFP binding partners and kinase activity by co-immunoprecipitation. (A) Top row: anti-GFP immunoblotting of CYCB1-GFP pull-down in untagged control (‘wt’), wt, *cdc27-6*, or *cdkb1-1* backgrounds. CYCB1-GFP was pulled down from lysates of cells that were first synchronized by nitrogen deprivation, then moved to restrictive temperature (33°C) in complete medium and collected after 13 hours. Typically, after 13 hrs. at 33°C, wild-type cells have undergone multiple fissions, and CYCB1-GFP level from a large population of cells is high (see, for example, the time course in S1 Fig, and microscopic images of wild-type in S5A Fig). Middle row: loading control is non-specific anti-GFP signal from immunoblot of total lysates before they were used for IP shown in the top row. Bottom row: kinase activity from immunoprecipitates shown in top row. All strains except for untagged wt control on left had temperature-sensitive *cycb1-5* rescued by *CYCB1*:*GFP-TG* transgene. Equal biomass based on OD_750_ was extracted and immunoprecipitated from each sample. Two independent strains were tested for each genotype. (B) Detection of CDKA1-mCherry or CDKB1-mCherry as possible binding partners of CYCB1-GFP. Strains with the indicated combinations of CYCB1-GFP and CDKA1-mCherry or CDKB1-mCherry were immunoprecipitated with anti-GFP from lysates of cells that were first synchronized by nitrogen deprivation, then moved to restrictive temperature (33°C) in complete medium and collected after 13 hours, a time when cells are mid-division phase based on prior experiments with this protocol. Input biomass was equalized by OD_750_. Immunoblotting was then done with anti-GFP or anti-mCherry. Two independent strains were tested for each genotype, such that lanes 7–12 reproduce the experiment in lanes 1–6. Loading control (bottom row) is non-specific protein staining from total lysates before immunoprecipitation.

Immunoblotting with anti-GFP revealed a band at the expected size for the fusion following immunoprecipitation (Fig 1A) or direct Western blotting (S1 Fig). Levels of CYCB1-GFP were low in newborn cells and rose as cells began division cycles, similar to the pattern seen with CDKB1 and the replication control proteins CDC6, ORC1, MCM6 and CDC45 ([29,68]; S1 Fig). This pattern of accumulation likely reflects strong transcriptional induction of these genes (along with essentially all other replication- or division-essential genes) in late G1 [43,44]. CYCB1-GFP signal declined to low levels late in the time-course as cells completed the multiple fission cycle; this decline was not observed in genetic backgrounds with the APC (*cdc27-6*, [29]) or CDKB1 (*cdkb1-1*, [28]) inactivated. CDC27 is a core APC subunit; *cdc27-6* is a temperature-sensitive lethal mutation in *CDC27* causing a tight first-cycle arrest [29]. *cdkb1-1* is a temperature-sensitive lethal mutation in the *CDKB1* coding sequence [28]. Although the wild-type cells in this experiment went through multiple division cycles, no oscillations of CYCB1-GFP levels were detected, only a single rise and fall. As we will show, this is due to imperfect synchrony of *Chlamydomonas*. There is no method of synchronizing bulk cultures that we know of that will give clean separations of cells at different positions in the multiple fission cycle (e.g., S phase of the third division). This is a major reason why single-cell methods are critical in this system.

Cyclins lack enzymatic activity on their own (see Introduction). Immunoprecipitated cyclins from cycling cells nevertheless can have associated protein kinase activity by virtue of tight binding and activation *in vivo* of a CDK. Histone H1 is a convenient *in vitro* substrate for enzymatic activity of many CDKs [1], including CDKB1 [29]. We immunoprecipitated CYCB1-GFP using anti-GFP nanobodies bound to magnetic beads. After washing immunoprecipitates and addition of 32P-ATP and histone H1, 32P-label accumulated on H1, indicating that protein kinase activity co-immunoprecipitated with CYCB1-GFP (Fig 1A). The signal was specific since it depended genetically on the *CYCB1*:*GFP-TG* transgene. In parallel we immunoprecipitated CYCB1-GFP from cells with the *cdc27-6* temperature-sensitive mutation, to inactivate the APC. Elevated levels of both CYCB1-GFP and co-immunoprecipitated kinase activity were observed. Approximate quantification of the Western blot compared to kinase activity suggested that most or all of the increased enzymatic activity detectable in the *cdc27-6* background was correlated to increased levels of CYCB1-GFP. We also immunoprecipitated CYCB1-GFP from cells with the *cdkb1-1* temperature-sensitive mutation, to inactivate CDKB1. CYCB1-GFP protein was detected in the immunoprecipitates, although at somewhat variable levels. Importantly, no kinase activity was observed in these immunoprecipitates, despite the presence of CYCB1-GFP protein (Fig 1A), suggesting that the histone H1 kinase activity co-immunoprecipitated with CYCB1-GFP in wild type cells was due to activated CDKB1. Reciprocally, we showed previously that CDKB1-associated kinase activity, but not CDKB1 protein levels, was strongly reduced in a *cycb1-6* background, inactivating CYCB1, and increased in *cdc27-6;CDKB1*:*mCherry-TG*. Taken together, these results suggest that regulation of CYCB1 abundance provides the major control of CDKB1 kinase activity.

We constructed *Chlamydomonas* CYCB1-GFP strains co-expressing CDKA1-mCherry or CDKB1-mCherry. Anti-GFP immunoprecipitation specifically co-precipitated CDKB1-mCherry but not CDKA1-mCherry (Fig 1B; results with two independent sets of congenic strains are shown), showing that CYCB1 binds specifically to CDKB1 but not CDKA1 *in vivo*.

Overall, these results are consistent with high specificity of CYCB1 for binding and activating CDKB1.

### Cyclin B accumulation and degradation during a multiple fission cycle

Increased accumulation of CYCB1-GFP in the absence of APC function (Fig 1A) suggested the likelihood that as in other systems, the APC promoted CYCB1 degradation. However, the imperfection of cell cycle synchrony in our hands is not good enough to resolve the individual rapid divisions, preventing determination of whether cyclin B was stable throughout the period of multiple fission and only degraded after all divisions were complete (as with MCM4 [68]), or was degraded in each division and then resynthesized.

To solve this problem, we developed methods for long-term time-lapse fluorescence microscopy of *Chlamydomonas*. This presented a number of technical challenges, including the need for highly accurate temperature regulation of cells being imaged (critical especially for temperature-sensitive mutants), preventing cells swimming out of the field of view, providing light for photosynthesis between image acquisitions, and computational subtraction of autofluorescence from chloroplasts, which otherwise swamped the CYCB1-GFP signal. Our solutions included small, individually sealed acrylic chambers filled with TAP/agarose, each containing a different cell population; overhead illumination provided by small LEDs, which were programmed to turn off transiently at the time of image acquisition; and computational deconvolution to eliminate contribution of chloroplast autofluorescence (Materials and Methods).

To our knowledge, the problems we solved (here and in [68]) had inhibited or blocked previous description by time-lapse-fluorescence imaging of the complete *Chlamydomonas* multiple fission cycle.

We also found that *Chlamydomonas* was quite sensitive to killing by illumination required for fluorescence imaging. For this reason, for long time-course movies, we limited exposure times and fluorescence intensity to the minimum required for detection, employed a 3-min frame-rate, and imaged only a single plane of focus that our autofocus routine placed at approximately the midline of the cell. This limited image intensity and resolution but allowed complete multiple fission cycles with essentially normal kinetics. Results reported here for these conditions were generally confirmed at least for the first division with 1-min or shorter frame-rates and multiple z-stacks with maximum projection (Methods; see below), which gave much higher-quality images but led to loss of cell viability after the first one or two divisions.

**Fig 2:**
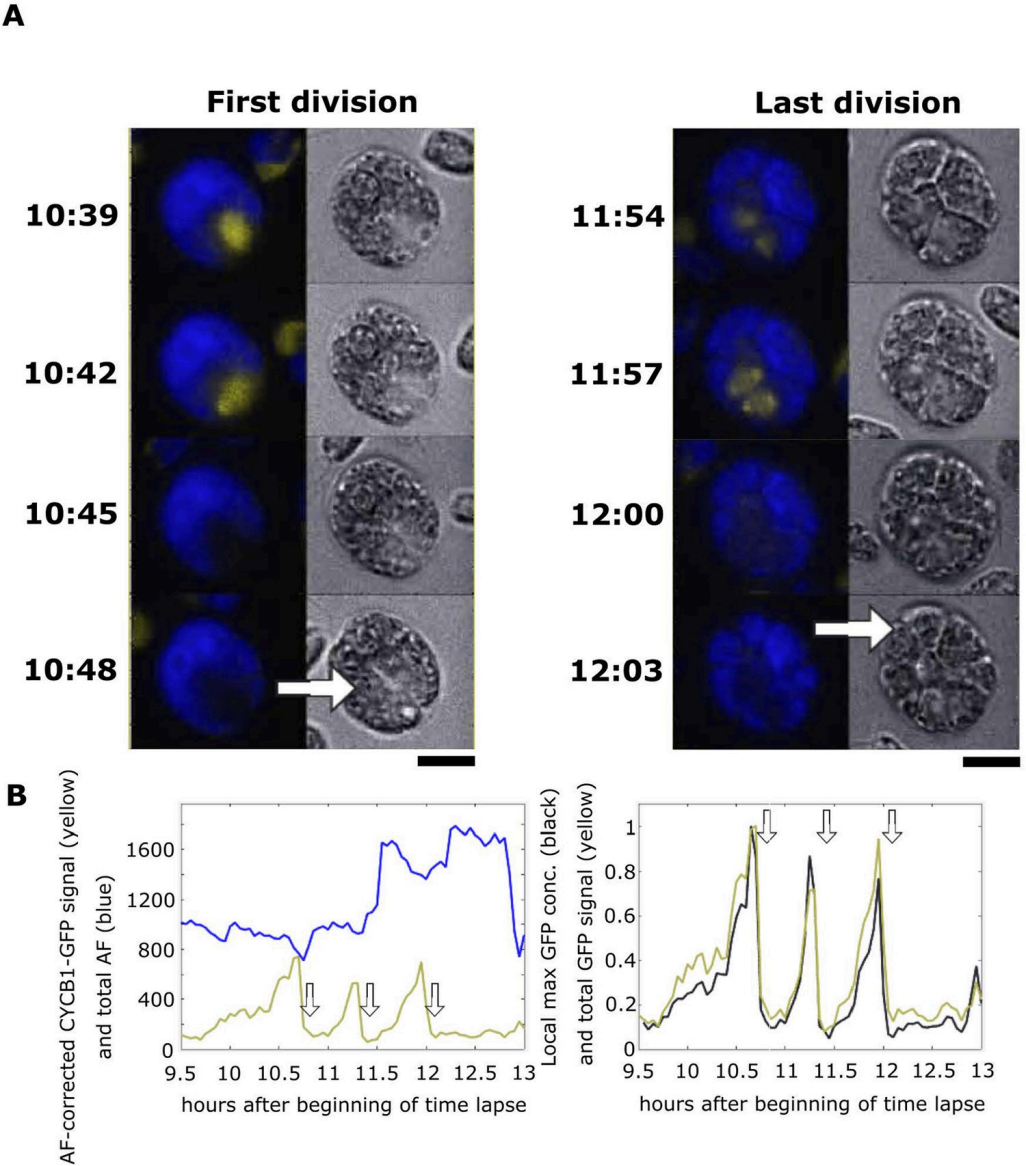
Live cell time-lapse microscopy of CYCB1-GFP. (A) Time-lapse images of CYCB1-GFP cells. Each cell has a brightfield image (right), and a composite of chloroplast autofluorescence in blue and CYCB1-GFP signal in yellow (left). Time indicated on top of each strip is hours and minutes after beginning of time-lapse. The indicated time corresponds to the top cell in each strip; cells below show the 3 min time-lapse series. Arrows indicate new cleavage furrow formation detected in brightfield. The imaged cell went through three divisions; frames surrounding the first and last divisions are shown. Scale bar: 5 microns. (B) Left: quantification of CYCB1-GFP signal deconvolved from chloroplast autofluorescence (yellow line), and chloroplast autofluorescence (blue line). Arrows: correspond to cleavage furrow formation. Right: Yellow trace: CYCB1-GFP total signal over the cell. Black: a minimal convex hull was computed that contained 50% of the CYCB1-GFP signal, and the concentration (signal/area) computed, showing that local concentration (probably within the nucleus; see text) and total cellular amount of CYCB1-GFP tracked closely through divisions. MATLAB code for calculating the convex hull available on request.

**Fig 3:**
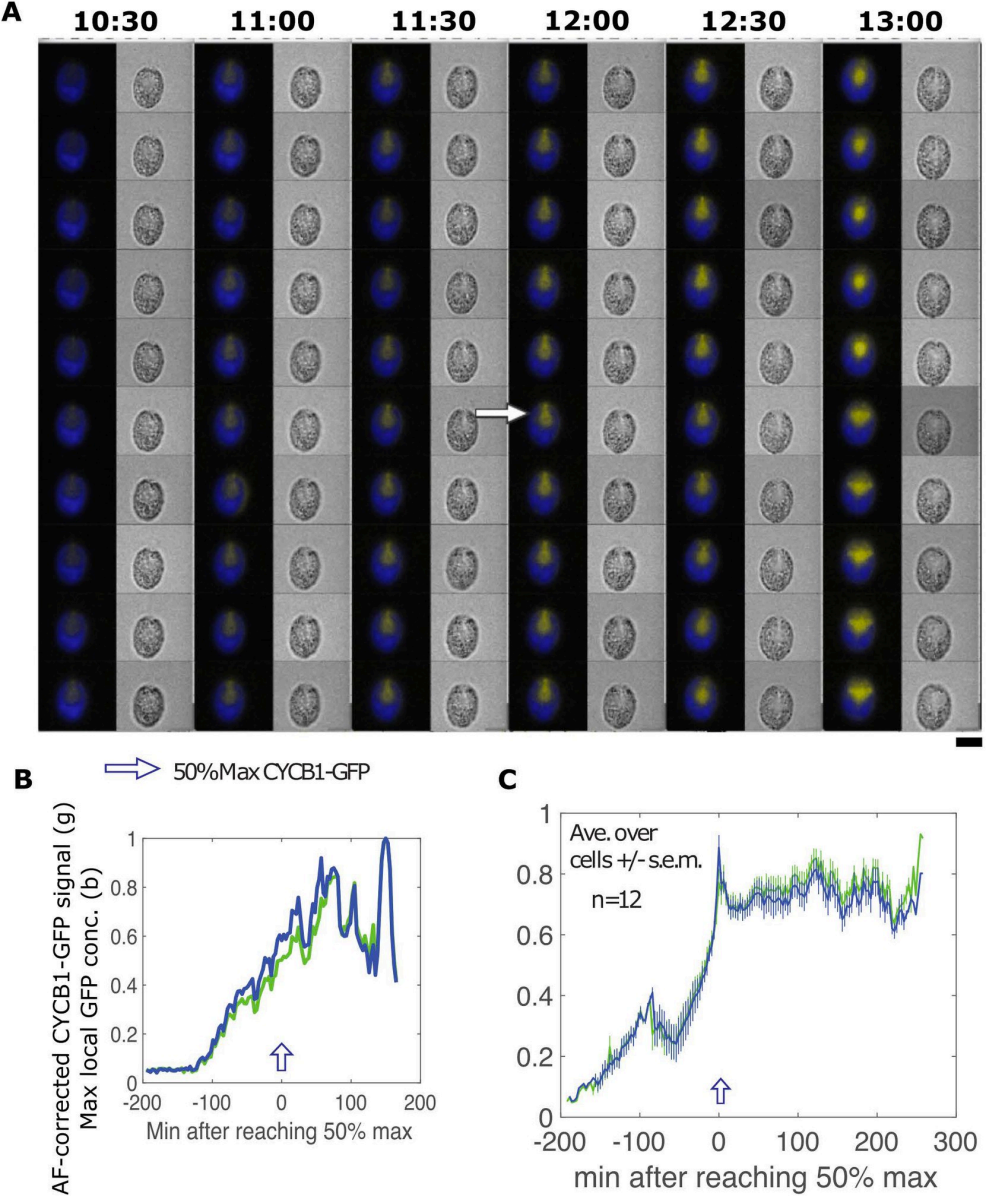
Live cell time-lapse of CYCB1-GFP in a *cdc27-6* background. (A) Each cell has a brightfield image (right), and a composite of chloroplast autofluorescence in blue and CYCB1-GFP signal in yellow (left). Time indicated on top of each strip is hours and minutes after beginning of time-lapse. The indicated time corresponds to the top cell in each strip. Each subsequent cell going down is from an image captured every 3 minutes. Scale bar: 5 microns. (B) Green: deconvolved total GFP signal in cell shown in A; blue: concentration estimated as in Fig 2. (C) The same plots for the average and s.e.m. of 12 cells. All traces adjusted to a maximum signal of 1 before averaging.

The computational deconvolution procedure (Methods; S2 Fig) was required for reasonably accurate quantification of CYCB1-GFP signal; however, the essential behavior was detectable even without deconvolution (S2 Fig). CYCB1-GFP signal was first clearly detectable over background ~0.5–1 hr before the first division (Fig 2 and S1 Video); signal increased steadily for approximately 20 min. From the shape and position of the signal we assume CYCB1-GFP is largely nuclear-localized, although we lack direct evidence for this. In some cells (especially those arrested by APC inactivation–see Fig 3 discussed below), we observe additional signals extending outside of the putative nuclear signal toward the cell anterior pole. We speculate that this anterior signal could have overlap with the centrin-containing nucleus-basal body connector [69].

After a period of steady accumulation, CYCB1-GFP signal dropped to near-background levels shortly before cell division (scored by formation of a cleavage furrow in a concurrent brightfield image; arrows in Fig 2 and S1 Video). From one typical movie at 3 min resolution, in which 15 individual cells could be imaged, we obtained values for CYCB1-GFP protein half-life during the period of rapid turnover of 3.0 +/- 1.3 min (mean +/- standard deviation; n = 15), and an interval of 1.2 +/- 2.4 min between reaching the trough in CYCB1-GFP levels to cell division. CYCB1-GFP then reaccumulates, but exclusively in cells that subsequently underwent an additional division (Fig 2), suggesting that the ‘decision’ to divide is upstream of CYCB1 accumulation. Additional movies with multiple strains gave highly similar results.

Newly accumulated CYCB1-GFP in later division cycles is sometimes clearly separated into 2 (2^nd^ division) or 4 (3^rd^ division) foci (Fig 2), which we presume corresponds to separate accumulation in different daughter nuclei. This is not always clearly observed; we think this is likely due to complexity of the multiply divided cells’ geometry observed at a single focal plane.

### Cyclin B proteolysis is dependent on APC and on CDKB1

APC-dependent ubiquitination and consequent proteolysis are frequently dependent on a ‘destruction box’ consensus sequence in the target protein [11]. CYCB1 contains a consensus destruction box [29]. Using the *cdc27-6* temperature-sensitive mutation in a core essential APC subunit [29], we found that CYCB1-GFP proteolysis was dependent on the APC (Fig 3): CYCB1-GFP levels were low at early times, and rose at similar times to wild-type, but unlike in wild-type, no precipitous degradation was observed even after many hours. Fig 3 shows results for 12 cells scored from a single movie; similar results were obtained in multiple additional experiments.

We also found that CYCB1-GFP levels did not undergo a precipitous drop in a *cdkb1-1* background, unlike in wild type (S3 Fig and S2 Video) (see Discussion).

### Is Cyclin B degradation essential

APC-dependent Cyclin B degradation is essential for mitotic viability in many but not all eukaryotic systems (Introduction).

We constructed a *CYCB1-db*:*GFP* transgene with the destruction box deleted, which is expected to confer insensitivity to APC-mediated proteolysis [11]. We transformed the transgene into a *cycb1-5* temperature-sensitive strain in parallel with wild-type *CYCB1*:*GFP*, selecting at 33 degrees for rescue of *cycb1-5*. In three independent experiments, each with large numbers of rescued colonies (too many to count, but more than 100 per plated transformation) using wild-type *CYCB1*:*GFP*, we obtained no colonies viable at 33 degrees upon electroporation with similar amounts of *CYCB1-db*:*GFP*, where *CYCB1* and *CYCB1-db* were in identical plasmid contexts and transformed at identical concentrations in parallel (Methods). This result is consistent with lethality of *CYCB1-db*. We cannot rule out the possibility that the CYCB1 destruction box is required for positive function of CYCB1; this has not been observed in other systems, however, and the destruction box is far from the cyclin regions responsible for CDK activation and substrate targeting. At any rate, these results indicate that the destruction box in CYCB1 is essential for its function in viable cells.

If *CYCB1-db* is truly lethal, it is predicted to be impossible to recover transformants expressing *CYCB1-db*, preventing study of the phenotype of the implied *CYCB1-db-*dependent lethality. The result is not simply a negative one, however, since we can calibrate failure to complement *cycb1-5* strains using *CYCB1-db* with the efficiency of recovery with *CYCB1-wt*. This problem was solved in budding yeast by conditional overexpression of the Sic1 stoichiometric inhibitor of cyclin B-CDK activity; this maintained viability of DB-deleted cyclin B strains, and the lethal phenotype could be studied when Sic1 was turned off [13]. Such a resource is not presently available in *Chlamydomonas*.

### Conservation of the anaphase-promoting role of the APC in *Chlamydomonas*

In yeast and animals, APC/CDC20 is required for sister chromatid separation and anaphase, due to the need to degrade the anaphase inhibitor securin to allow ESP1 activation and cleavage of the cohesin complex, and this is independent of the role of APC/CDC20 in removal of cyclin B (see Introduction). In the plant kingdom, a securin homolog was only recently identified [50], and while APC subunits are clearly essential in land plants (see Introduction), the cytological consequences of APC inactivation are less clear.

**Fig 4:**
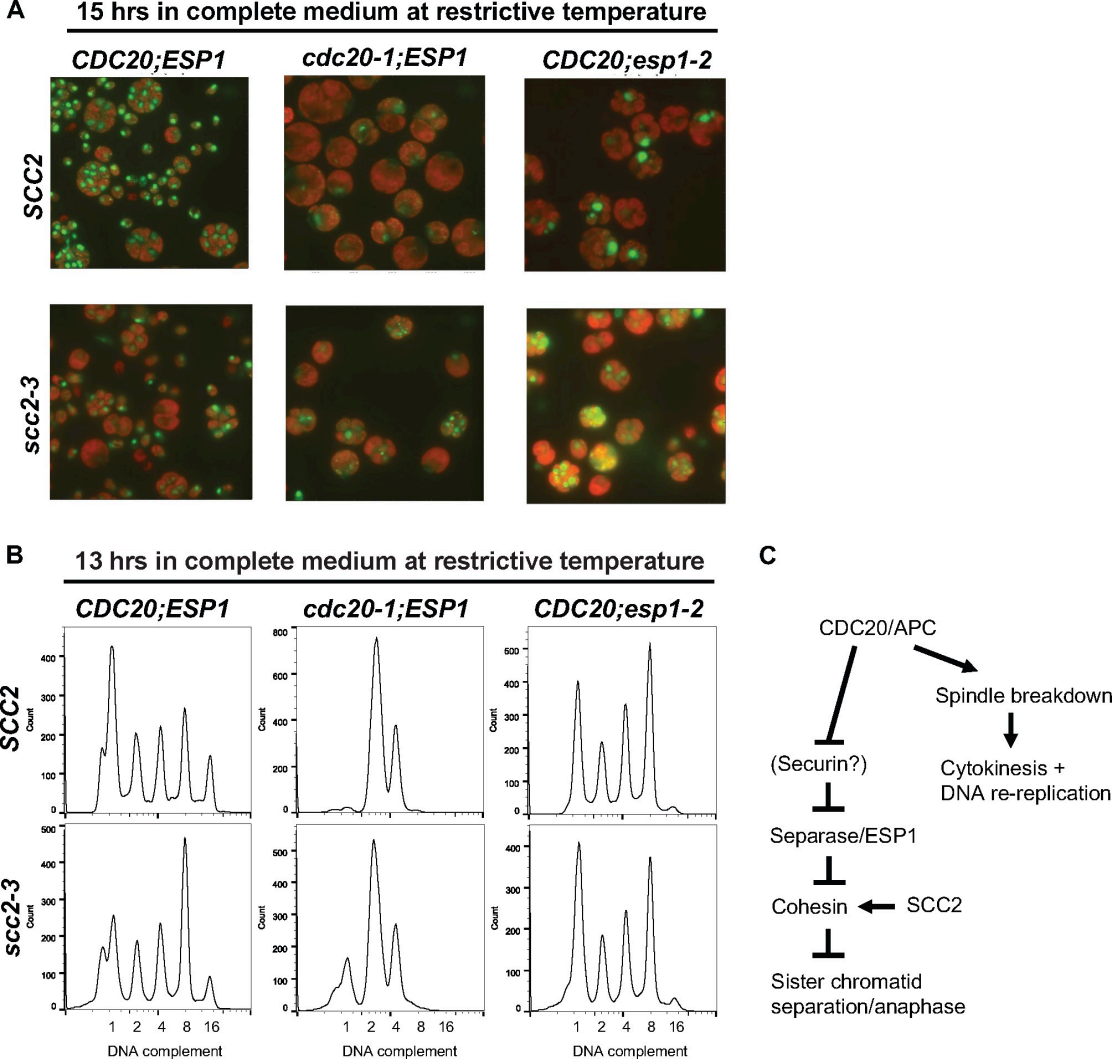
DNA localization and replication in APC (*cdc20*) and separase (*esp1*) mutants. (A) Wild-type, *cdc20-1*, *esp1-2* mutants with or without *scc2-3* were blocked in G1 by nitrogen deprivation, as described [29], then placed at restrictive temperature (33°C) in complete medium, collected after 15 hrs., and stained with Syto11 live cell green fluorescent nucleic acid stain. Red signal is from chloroplast auto-fluorescence. (B) Flow cytometry from same set of cells as (A) but after a collection at 13 hrs. For flow cytometry data from 0 and 15h, as well as corresponding cell size data, see S4A Fig. (C) Proposed model for the regulation of both DNA replication and sister chromatid separation by CDC20/APC.

Previously, we isolated mutations in many components of this system: APC subunits, CDC20, the cohesin loader SCC2, and separase/ESP1 [28,45]. We characterized the cell cycle arrests of single and double mutants in this set. CDC20 inactivation resulted in arrest with once-replicated DNA (2C DNA by flow cytometry, with sporadic escapers progressing to 4C), and live-cell staining of DNA with the dye Syto11 revealed a single DNA mass without cytokinesis (Fig 4A). ESP1 inactivation also resulted in a single DNA nucleus, in which the DNA staining signal was much brighter than with CDC20 inactivation. Consistently, flow cytometry of arrested *esp1* mutants showed they underwent at least three complete and sequential rounds of DNA replication, accumulating with 8C DNA content (Figs 4B and S4A). Cells also exhibited aberrant cytokinesis, with segregation of the single large nucleus into one of the cell bodies ([28]; Fig 4A). The DNA content results indicate a requirement for APC/CDC20, but not for ESP1 or sister chromatid separation, for a new round of replication to occur. SCC2 inactivation, which is expected to interfere with loading of the cohesin complex, had little or no effect on the replication profile. *scc2* cells appeared also to undergo a cytologically normal multiple fission cycle, although the resulting daughter cells were mainly or entirely inviable [45]. Double mutants in this set were also analyzed. *cdc20;esp1* double mutants were, for some *esp1* alleles, inviable in tetrad analysis, consistent with CDC20 and ESP1 acting in the same pathway; but with other alleles the double mutant is viable, and when arrested at high temperature is indistinguishable morphologically from the *cdc20* single mutant (S5A Fig), in particular in the lack of detectable cytokinesis. *cdc20;esp1* double mutants arrested with 2C DNA content like *cdc20* single mutants (S6A Fig). *cdc20;scc2* and *esp1;scc2* double mutants had DNA content by flow cytometry similar to *cdc20* and *esp1* single mutants respectively (Fig 4B), but notably, the single large DNA masses observed in *cdc20* and *esp1* single mutants were replaced with variable numbers and sizes of smaller masses (Fig 4A). This is consistent with at least a partial relief of sister chromatid cohesion in the double mutants due to SCC2 inactivation. In addition, we noted a partial rescue of ts-lethality due to one *esp1* allele when combined with *scc2-3* (S5B Fig). These results overall are consistent with a branched pathway with CDC20/APC at the top regulating two separate branches, one leading to DNA replication and cytokinesis, and another to sister chromatid separation; ESP1, SCC2 and a hypothetical securin analog might participate only in the branch regulating sister chromatid separation (Fig 4C). We observe a highly similar arrest phenotype (high-ploidy mononucleated cells) with 5 independent *esp1* alleles, and the causative mutations in these alleles are found in multiple different regions of the *ESP1* coding sequence (S4 Fig). Therefore, we think this is the true null phenotype for *esp1* in *Chlamydomonas*, and separase may be required for sister separation but not for mitotic exit, including cytokinesis and additional rounds of DNA replication. In this way *Chlamydomonas* may resemble animal cells rather than budding yeast ([17]; see Introduction). This implies that the sister-separation role of separase, but perhaps not other roles, was established already near the time of the last eukaryotic common ancestor [21].

### Regulation of CDKB1

**Fig 5:**
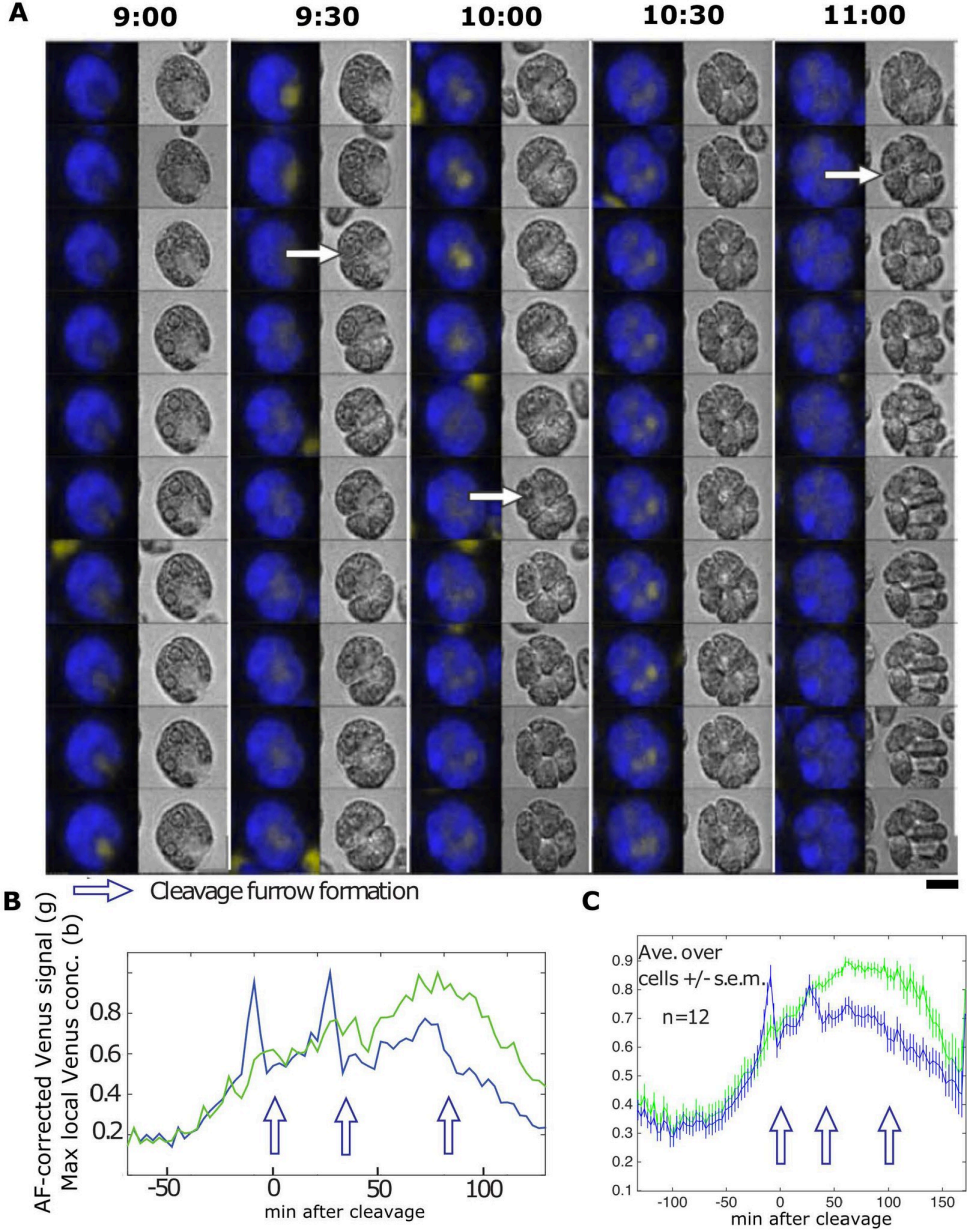
Live cell time-lapse of *CDKB1*:*Venus-TG* cells. (A) Brightfield image (right), and a composite of chloroplast autofluorescence in blue and CDKB1-Venus signal in yellow (left). Time indicated on top of each strip is hours and minutes after beginning of time-lapse. The indicated time corresponds to the top cell in each strip. Each subsequent cell going down is from an image captured every 3 minutes. Arrows: cleavage furrow formation. Scale bar: 5 microns. (B) Quantification of YFP signal in the cell shown in (A). Green line: total YFP signal; blue line: estimated concentration (YFP signal per area) in the minimal convex hull calculated to contain 50% of total signal. (C) The same plots for the average and s.e.m. of 12 cells. All traces adjusted to a maximum signal of 1 before averaging. In B and C, note reproducible peak of concentration of CDKB1-Venus 1–2 frames before cleavage furrow formation. This pattern repeated in successive cycles; the peak was reduced in intensity in the averaged data, most likely due to slight asynchrony in timing comparing different cells.

We reported previously that CDKB1-mCherry accumulated in the nucleus of cells during the multiple fission period [29]; however, using bulk culture measurements, we were unable to resolve whether CDKB was degraded and then resynthesized in each cell cycle, as suggested based on results in *Arabidopsis* and *Ostreococcus* [32,37]. We could not answer this question by time-lapse microscopy of *CDKB1*:*mCherry-TG* cells, because the spectral overlap of mCherry and chloroplast autofluorescence was too great for the deconvolution method to handle with the filters we had available, given relatively low CDKB1-mCherry signal [29]. Therefore, we constructed a *CDKB1*:*Venus* transgene, and used it to rescue a *cdkb1-1* strain. *Chlamydomonas* autofluorescence under Venus detection was low enough to reliably detect signal from CDKB1-Venus (Fig 5). In time-lapse microscopy of rescued cells, we observed CDKB1-Venus accumulation only during the period of the multiple fission cycle, consistent with previous results [29]. However, quantification following computational deconvolution (as described above; Methods) showed that there was no loss of total CDKB1-Venus signal at any point during the individual divisions, unlike the behavior of CYCB1-GFP (Fig 5). Since CYCB1-GFP and CDKB1-Venus images were deconvolved and processed identically (Methods), we conclude that CYCB1-GFP cycles in abundance in each cycle, but CDKB1-Venus does not.

Although CDKB1-Venus signal quantified over the entire cell remained high through multiple division cycles, the local intensity of the nuclear signal varied through the cell cycle, reaching a peak about 6 min before division (Fig 5). The timing of more intense CDBK1-Venus localization approximately corresponds to the timing of CYCB1-GFP accumulation. We speculate that efficient nuclear localization of CDKB1 may require CYCB1.

Quantitatively reproducible behavior of 12 cells from a single movie is shown in Fig 5; additional movies with multiple highly backcrossed *cdkb1-1* strains gave consistent results. After completion of the terminal cell division, CDKB1-Venus remained diffuse and disappeared over the succeeding ~1 hr (Fig 5), suggesting that CDKB1 degradation might be dependent on exit from the multiple fission period.

### Live-cell imaging with EB1-mNeonGreen reveals regulation of microtubule structures as cells enter mitosis

The microtubule end-binding protein EB1 marks the plus ends of growing microtubules, and provides a useful marker for microtubule-dependent mitotic events in many systems including *Chlamydomonas* (Introduction). We used two different imaging methods to precisely record the behavior of EB1-NG in dividing wild-type and mutant cells. In method 1, we used 3-min intervals with single Z-planes to avoid phototoxicity, and the precise temperature control to image ts-lethal mutants through multiple division cycles, as described above in detail. Method 1 was also used for a short movie with 20-sec intervals. In method 2, we used 10-sec intervals with single Z-planes to examine EB1-labeled structures that are near the medial plane (anterior spots, spindle, and furrow) in the first division of a multiple fission cycle [57].

**Fig 6:**
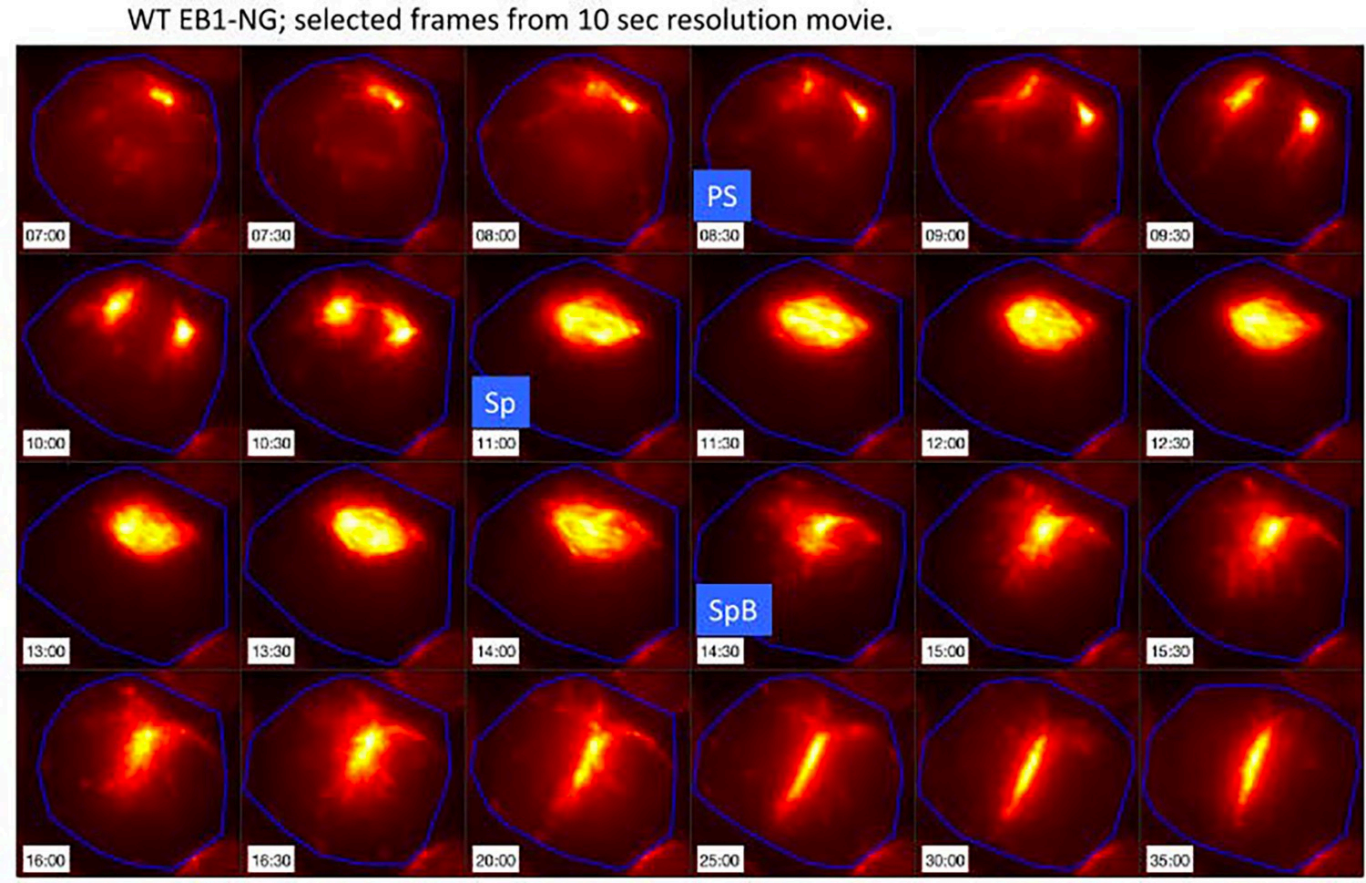
Live cell time-lapse of wild-type *EB1*:*NG-TG* cells with 10-sec. intervals. Live cell time-lapse with 10-sec. intervals acquired with microscopy method 2 (see Methods section). EB1-NG signal in orange. PS: pole separation. Sp: spindle formation. SpB: spindle breakdown.

**Fig 7:**
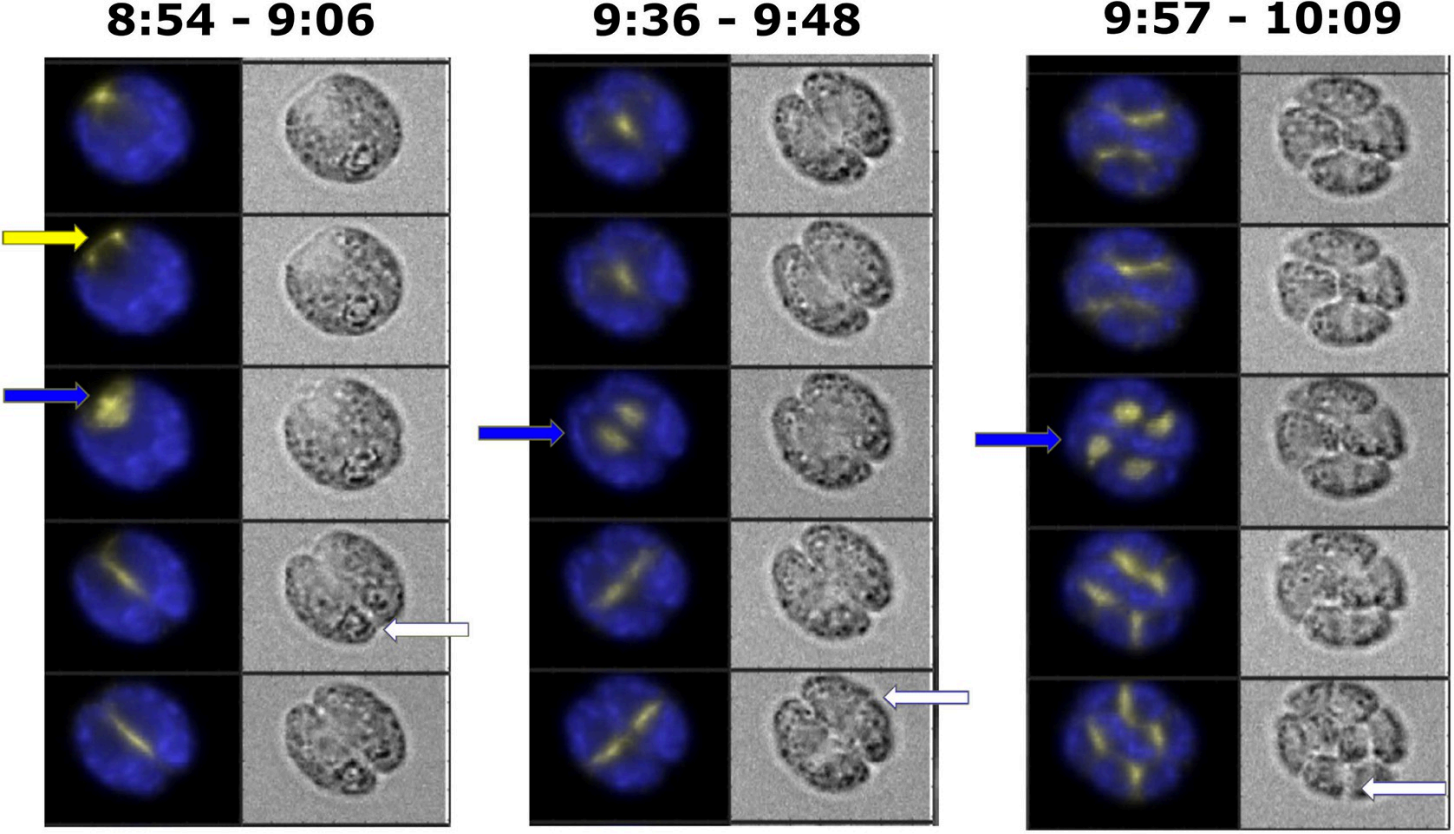
Live cell time-lapse of wild-type *EB1*:*NG-TG* cells with 3-min. intervals. Live cell time-lapse with 3-min. intervals acquired with microscopy method 1 (see Methods section). Each cell has a brightfield image (right), and a composite of EB1-NG signal in yellow and chloroplast autofluorescence signal in blue (left). Yellow arrow: spindle pole separation. Blue arrows: new spindle formation. White arrows: new cleavage furrow formation. Time indicated on top of each strip is hours and minutes after beginning of time-lapse. The indicated time corresponds to the top cell in each strip. Each subsequent cell going down is from an image captured every 3 minutes.

As cells enter mitosis, the polar ‘spot’ of EB1 signal splits into two; the two spots move slightly into the cell interior and mark foci that nucleate formation of a bipolar spindle about 4 min after pole splitting (Figs 6 and 7 and Table 1 and S3 and S4 Videos). Note that we use the terms ‘polar’, ‘poles’ and ‘pole splitting’ without implication as to their relationship to basal bodies, spindle poles or other known microtubule-based mitotic structures. In *Chlamydomonas*, basal bodies and spindle poles are near to each other but spatially distinct [70]. Determining these relationships requires multiple labeling and probably would also require super-resolution microscopy, which is technically challenging and incompatible with time-lapse, at least with our present methods.

**Table 1:** Timing of EB1-scorable mitotic events. Time-lapse movies at varying frame-rates (top row) were analyzed manually, and mean and standard deviation of time intervals is presented (all in minutes). ‘Pole sep’: separation of the anterior EB1 signal into two separate foci. ‘SP1, SP2, SP3’: full formation of bipolar spindle in 1^st^, 2^nd^, 3^rd^ rounds of division. A spindle was scored when the EB1-NG signal was continuous across the midline of the cell, and the orientation of the signal was roughly perpendicular to the following cleavage furrow. ‘SP1B, SP2B, SP3B’: spindle breakdown 1^st^, 2^nd^, 3^rd^ rounds of division. Spindle breakdown was scored when the EB1-NG signal was no longer perpendicular to the following cleavage furrow. Spindle formation and breakdown were highly synchronous in progeny within a single cell, although not all spindles were in focus in every division. To calculate the spindle duration, or SP to SPB, the frame number of the first visible spindle was subtracted from the frame number of the start of the spindle breakdown, and then multiplied by the frame frequency. In cases where a spindle was not visible in any frame it was given a value of 0 frames, but only if the preceding PS was clearly visible; in these cases, it was assumed that the spindle was likely missed because it was not present at the times the images were captured. If neither the PS nor the SP were seen, the values were not recorded. ‘CF1, CF2, CF3’: detectable cleavage furrow initiation in 1^st^, 2^nd^, 3^rd^ rounds of division (for later divisions, furrow formation in any progeny cell was counted due to image complexity). A CF was scored when a visible indent associated with a dark line in brightfield, aligned with the upcoming plane of separation of the cells. The 10-sec frame-rate movie was at 26°C; the 20-sec and 3-min movies were at 33°C. Entries are in minutes: mean +/- standard deviation (number of cells). ND: not determined, for the following reasons: (1) 10 and 20 sec frame-rates were only usable for the first division, as cell viability dropped from light exposure; (2) cleavage furrow formation was scored from brightfield images that were captured only at 3 min resolution. Effective detection of pole separation was difficult at 3 min resolution because it was easiest to observe when moving density could be compared between adjacent frames. Only the later phases of the PS were easily seen once the poles were separated and very bright. Therefore, this was not scored in the 3 min frame-rate movies. See Figs 6 and 7 and S3 and S4 Videos for illustrative examples.

	Frame-rate		
Event	10 sec	20 sec	3 min
Pole sep → SP1	3.3 (1)	4 +/- 1 (14)	ND
SP1 → SP1B	3.8 (1)	3.7 +/- 0.7 (16)	3 +/- 1 (30)
SP1B → CF1	ND	ND	1 +/- 1 (24)
SP1 → SP2	ND	ND	37 +/- 3 (18)
SP2 → SP2B	ND	ND	2 +/- 1 (28)
SPB2 → CF2	ND	ND	2 +/- 2 (20)
SP2 → SP3	ND	ND	41 +/- 5 (15)
SP3 → SP3B	ND	ND	3 +/- 1 (25)
SP3B → CF3	ND	ND	2 +/- 2 (23)

S3 Video shows the process of pole splitting, spindle formation, anaphase and cytokinesis all marked by EB1-NG, at 10-sec time resolution in the first division cycle. S4 Video, at 3-min resolution, shows the same sequence repeating in three sequential divisions. As noted in Methods, the 10-sec resolution resulted in sufficient irradiation of the cells that after the first division, additional divisions were rarely observed. Imaging only every 3 min allowed multiple rapid divisions (Figs 6 and 7 and S3 and S4 Videos).

The spindle structure, as detected by EB1 signal, has a ~4 min lifetime, then disappears; signal remains at approximately the position of the spindle midzone, and this signal rapidly elongates perpendicular to the spindle axis (Figs 6 and 7 and S3 and S4 Videos). This line of EB1 signal is detected coincident with a cleavage furrow (detectable in a paired brightfield image), perpendicular to the former spindle axis. EB1 signal and the cleavage furrow extend essentially together in space and time (Fig 7).

Since formation of a cleavage furrow detectable in brightfield almost invariably occurs in the 3-min frame following spindle detection (Table 1), and CYCB1-GFP degradation tightly correlates with cleavage furrow formation (Fig 2; see above), these combined results suggest near-simultaneous spindle breakdown and CYCB1 degradation followed by cleavage furrow formation.

In multiple fission, additional cell division cycles occur within the same mother cell wall [40]. These cycles are rapid and regularly spaced (Table 1). S4 Video shows the high degree of synchrony of successive divisions within the descendants of a given mother cell, and the reliable appearance of the cytokinetic furrow at right angles to the long axis of the spindle immediately after spindle breakdown.

We almost invariably observe simultaneous appearance in the second division of two bipolar spindles, which disappear in the next frame replaced by two lines of EB1 signal perpendicular to the long axes of the spindles. In most cells, cleavage furrows were detectable in that same frame (Fig 7 and S4 Video). In favorable 3^rd^-division cells, similar observations can be made of four bipolar spindles (Fig 7 and S4 Video). We expect that this reflects similar microtubule and EB1 behavior in the later divisions to what was observed in the first. However, 3-min frame resolution is close to the interval between pole splitting and spindle formation, and between spindle formation and breakdown; as a consequence, in a sizable fraction of cells we observe pole splitting or spindle formation, but not both.

We proposed above that in *Chlamydomonas*, ESP1 functions specifically in regulation of sister chromatid cohesion, and lacks additional roles proposed for it in other organisms (see above and Introduction). To test this further, we carried out time-lapse of an *esp1;EB1*:*NG-TG* strain at non-permissive temperature (S5 Video). In the first cell cycle these cells presented EB1-NG behavior generally similar to wild type: pole splitting, spindle formation and breakdown and formation of the cytokinetic furrow. (Later cycles are aberrant and difficult to interpret, consistent with the highly aberrant morphologies already demonstrated for *esp1* mutant cells after several attempted divisions [Figs 4A and S5A]). But the apparently normal execution of a complete pole-to-spindle-to-furrow EB1 cycle in the first division suggests that ESP1 is not required for any of these events, consistent with its specific involvement in sister cohesion but not in other aspects of spindle control, resembling results in mouse cells [17].

The results above provided us with a clear picture of the events and timing of EB1-labeled mitotic events in wild-type. Comparison to timing of CYCB1-GFP accumulation suggested a correlation between CYCB1 presence and ‘pole splitting’ and spindle formation, and between CYCB1-GFP disappearance and spindle breakdown and cytokinesis. To determine if these correlations reflected causality, we determined the EB1-NG phenotype of mutants either blocking CYCB1-CDKB1 function (*cycb1-5* or *cdkb1-1*) or blocking CYCB1-CDKB1 inactivation (*cdc27-6*, *cdc20-1*). All results with mutants were confirmed with multiple isolates of multiply backcrossed strains.

**Fig 8:**
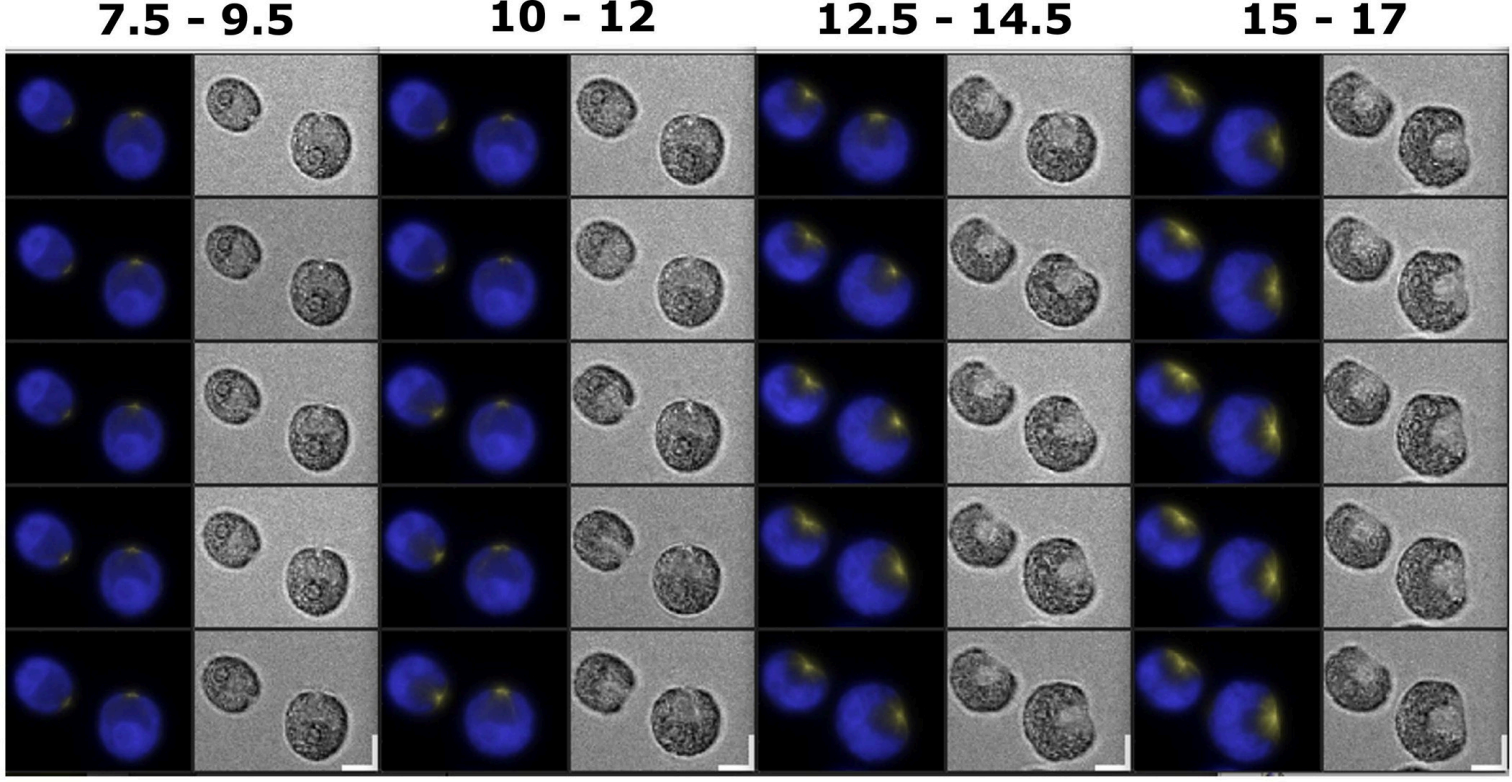
Live cell time-lapse of *cycb1-5*;*EB1*:*NG-TG* cells. Live cell time-lapse acquired with microscopy method 1 (see Methods section). Each cell has a brightfield image (right), and a composite of EB1-NG signal in yellow and chloroplast autofluorescence signal in blue (left). Time indicated on top of each strip is hours after beginning of time-lapse. The indicated time corresponds to the top cell in each strip. Each subsequent cell going down is from an image captured every 30 minutes.

In *cycb1-5* cells expressing EB1-NG (Fig 8 and S6 Video), we observed only the anterior spot of EB1-NG signal, as in wild-type G1 cells, even at long incubation periods (when wild-type cells have almost all divided). The polar splitting event, and subsequent spindle formation, were not observed. This is consistent with the correlative result that polar splitting and spindle formation follow CYCB1 accumulation. Spindle formation detected by anti-tubulin immunofluorescence was previously shown to be defective in *cdkb1-1* and *cycb1-5* cells [28,29]. The present results show that even early steps in spindle morphogenesis do not occur in these mutant backgrounds. This contrasts with the essentially normal induction of transcription of the mitotic gene cluster in the absence of CDKB1 function [43], which includes many genes likely involved in spindle morphogenesis. This contrast suggests specific requirements for CYCB1-CDKB1-dependent phosphorylation for spindle formation, such as CDK phosphorylation of the kinesin motor Eg5 in yeast and animals [71].

Reciprocally, we noted above that the timing of CYCB1 degradation was very close to the timing of spindle breakdown (compare Fig 2 to Table 1). To ask if this correlation might reflect a causal relationship, we blocked CYCB1 removal by inactivating the APC using the *cdc27-6* mutation (inactivating an essential APC core subunit). We also tested APC/CDC20 inactivation with the *cdc20-1* mutation.

**Fig 9:**
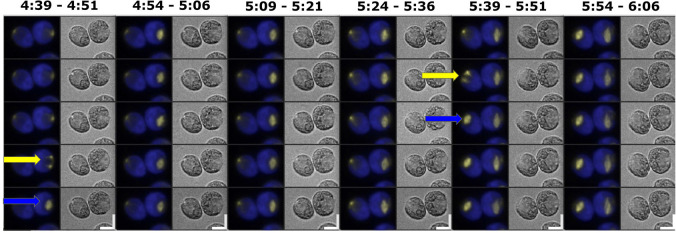
Live cell time-lapse of *cdc27-6;EB1*:*NG-TG* cells. Live cell time-lapse acquired with microscopy method 1 (see Methods section). Each cell has a brightfield image (right), and a composite of EB1-NG signal in yellow and chloroplast autofluorescence signal in blue (left). Yellow arrows: spindle pole separation. Blue arrows: new spindle formation. Time indicated on top of each strip is hours and minutes after beginning of time-lapse. The indicated time corresponds to the top cell in each strip. Each subsequent cell going down is from an image captured every 3 minutes.

**Fig 10:**
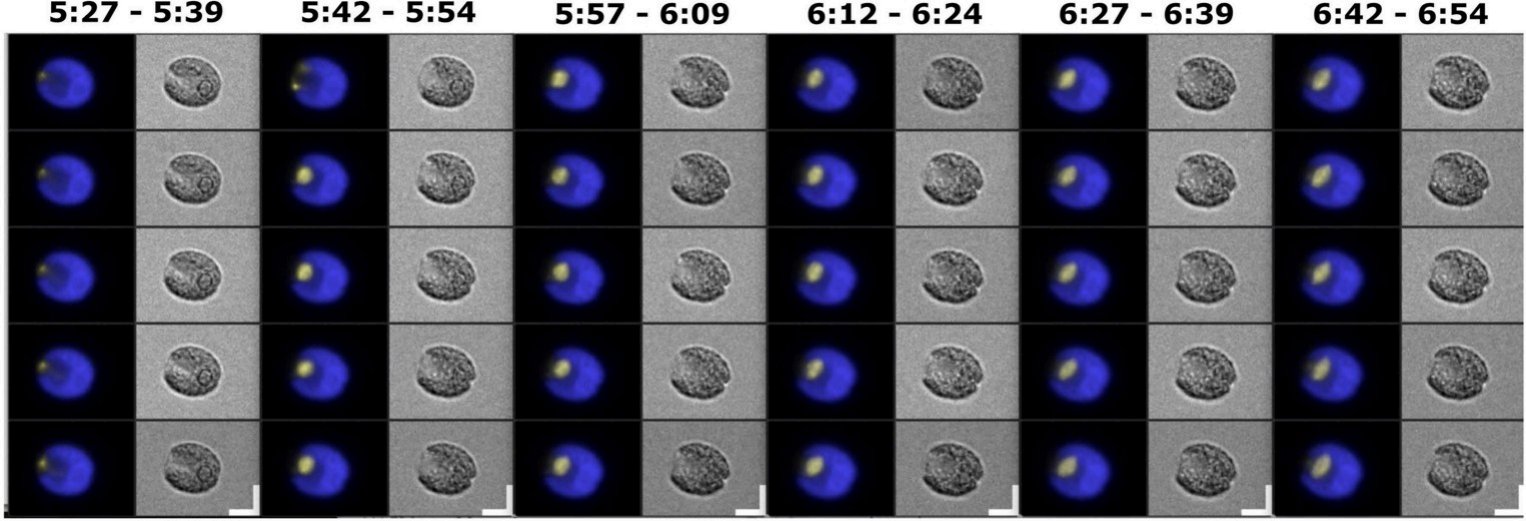
Live cell time-lapse of *cdc20-1;EB1*:*NG-TG* cells. Live cell time-lapse acquired with microscopy method 1 (see Methods section). Each cell has a brightfield image (right), and a composite of EB1-NG signal in yellow and chloroplast autofluorescence signal in blue (left). Time indicated on top of each strip is hours and minutes after beginning of time-lapse. The indicated time corresponds to the top cell in each strip. Each subsequent cell going down is from an image captured every 3 minutes.

In both mutant backgrounds, cells expressing EB1-NG at restrictive temperature underwent the polar splitting reaction followed by efficient bipolar spindle formation (Figs 9 and 10, and S7 and S8 Videos). Once formed, the spindle was stable, lasting for many hours (in contrast to ~4 min in wild type), consistent with previous observations with anti-tubulin immunofluorescence [29]. No cytokinetic cleavage furrow formed; correlated to this, there was no EB1-NG signal aligned perpendicular to the spindle long axis.

While APC-CDC20 is required for sister chromatid separation due to the need for securin breakdown leading to cohesin cleavage, spindle breakdown does not require either of these events in yeast and animals [10]; therefore, we propose that the most likely target responsible for spindle maintenance in the absence of APC is CYCB1.

**Fig 11:**
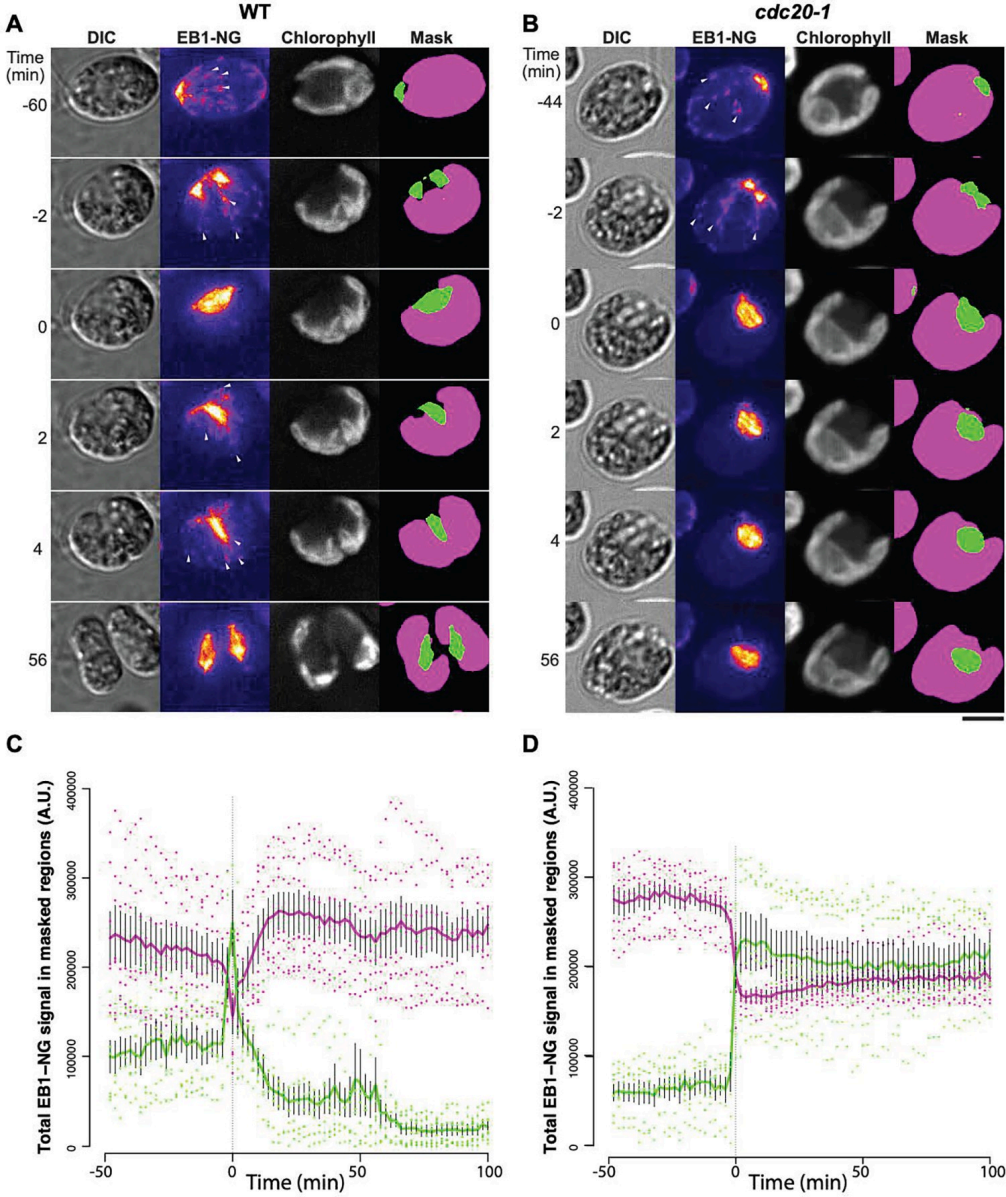
Suppression of cytoplasmic EB1-NG comets during mitosis. (A, B) Representative examples of wild-type (A) and *cdc20-1* (B) cells expressing EB1-NG. Time-lapse microscopy was done using Method 3 (see Materials and Methods). Bar, 5 μm. (C, D) Regions representing polar dot, spindle, and furrow (green in A, B) and cytoplasm (magenta in A, B) were masked as described in Materials and Methods. Total signals in the masked regions are presented as mean ± SEM (N = 7), with values from individual cells overlaid as dots. Time zero (first appearance of complete spindle) was determined empirically for each cell. See S7 Fig for individual traces.

In interphase, EB1-NG is associated with ‘comets’ that move from the anterior along the cortex to the posterior [55], presumably marking rapid microtubule growth. These comets disappear for a very brief interval exactly coincident with the presence of a spindle (Figs 11A and 11C and S7). In *cdc20-1* cells the spindle is stable, and comet suppression is permanent (Fig 11B and 11D). This suggests APC-Cdc20-dependent degradation of an inhibitor of comet formation.

It is interesting to note that redistribution of EB1 from comets to the vicinity of spindle poles as cells enter mitosis was also characterized in *Arabidopsis* [66,67], suggesting that this redistribution may be conserved through *Viridiplantae* (algae and land plants). The specific involvement of cyclin-Cdk in this redistribution in *Arabidopsis* is unclear, but deletion of subsets of the 12-member cyclin B family was shown to interfere with accurate and timely mitotic spindle formation [51]. The gamma-tubulin ring complex-binding protein GIP1 was suggested as a CDKB phosphorylation target that could account for this requirement [51]. *Chlamydomonas* has a clear GIP1 homolog (Cre14.g610000), but this protein lacks the proposed CDK phosphorylation site found in the *Arabidopsis* protein.

### Simultaneous timelapse microscopy of CYCB1 and EB1

**Fig 12:**
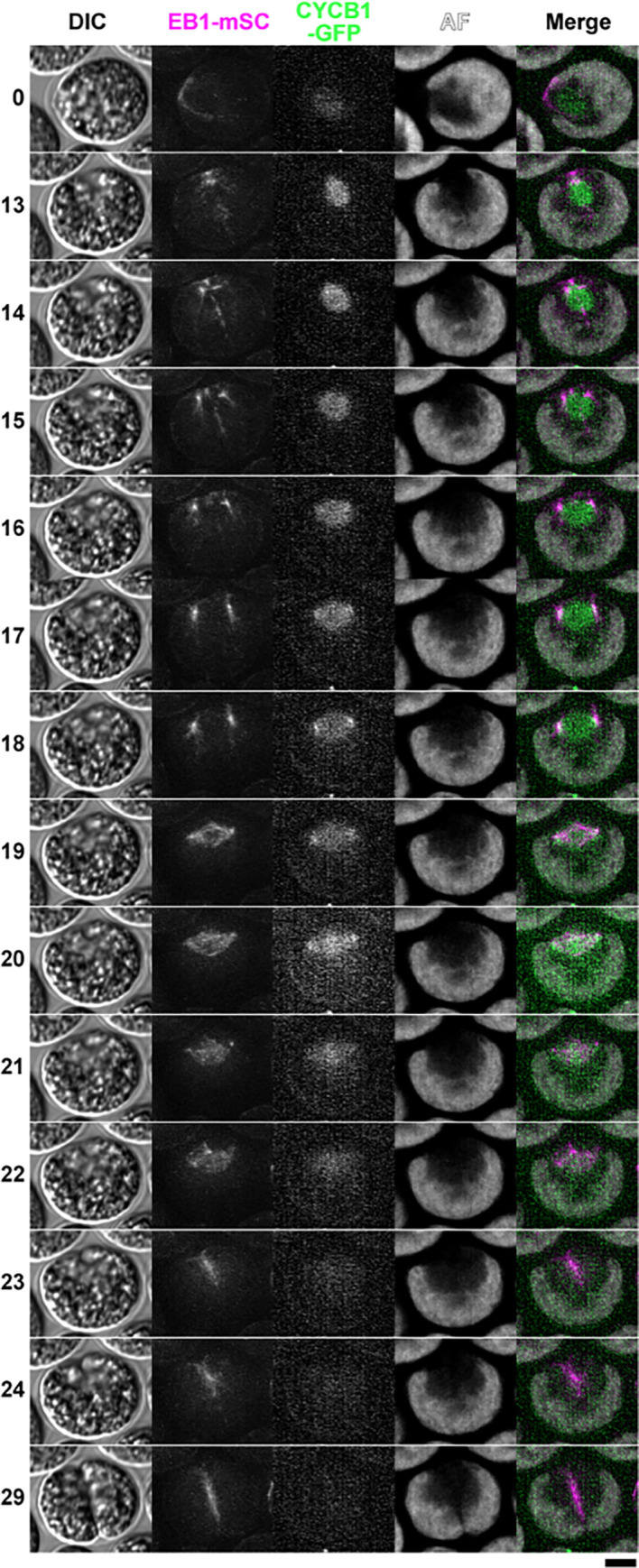
Live cell time-lapse of *CYCB1*:*GFP;EB1*:*mScarlet-TG* cells with 1-min intervals. Live cell time-lapse imaging was done using Method 3 (see Methods section). Imaging was done at 27°C. Select time-frames are shown with the times indicated on the left (min.). For DIC and AF (chlorophyll autofluorescence), mid-section images are shown; for EB1-mSc, maximum projections of Z-stacks are shown; for CYCB1-GFP, maximum projections of Z-stacks after Gaussian blurring along the Z-axis are shown. Bar, 5 microns. See S9 Video for a larger field of view including this cell through the entire time-series. In this experiment, we used an allele of *CYCB1*:*GFP* in which the endogenous CYCB1 locus was tagged with GFP; results with the endogenously tagged CYCB1 were in general very comparable to results with the transgene used elsewhere in the paper.

To examine spatial and temporal correlations between CYCB1 and EB1, we constructed a *CYCB1*:*GFP EB1*:*mScarlet* strain, and observed the first division with live cell microscopy (microscopy method #3 with z-stacks). The localization of EB1-mScarlet is similar to what was observed above with EB1-NG: a polar signal splits into two as cells enter mitosis, and a bipolar spindle is formed from these two spots; the spindle then disappears and EB1 collects along the cleavage furrow (Figs 6 and 12; [57]).

CYCB1-GFP accumulates steadily with a presumed intra-nuclear localization (Fig 12, S9 Video). Just before spindle formation, CYCB1-GFP signal is briefly concentrated at or just adjacent to the EB1 foci (Fig 12, timepoints 18–20 min; S9 Video). This implies that CYCB1-GFP localizes at or near the future spindle poles. As the spindle breaks down, CYCB1-GFP shows transient localization to a band at the approximate location of the former spindle midzone; CYCB1-GFP is then completely degraded. Thus, CYCB1 degradation and spindle breakdown are nearly simultaneous, in agreement with our conclusions comparing separate CYCB1-GFP and EB1-NG movies (see above).

The genetic requirement for CYCB1 for spindle formation, and the localization of CYCB1 to spindle poles just before spindle formation, suggests the speculation of direct regulation of spindle assembly by pole-localized CYCB1.

## Discussion

### CYCB1 interacts with CDKB1

Previously [29], we inferred that CYCB1 was the most likely activator of CDKB1 enzymatic activity, since CDKB1-associated histone H1 kinase activity was greatly reduced in immunoprecipitates from *cycb1-5* cells. Here, we confirm and extend this finding: CYCB1 binds CDKB1 but not CDKA1 in immunoprecipitates from doubly tagged cells, and CYCB1-associated histone H1 kinase activity is absent in immunoprecipitates from *cdkb1-1* cells.

Specificity of interactions between cyclins and CDKs in *Arabidopsis* has been inconclusive. Comprehensive proteomics with tagged proteins showed that cyclin B bound specifically to CDKB and not CDKA [72]; however, Boruc et al. [73] showed by binary interaction assays that CDKB and CDKA both have the capacity to bind CYCBs and CYCAs. Motta et al. [51] showed preferential interaction of *Arabidopsis* CYCBs and CDKBs to make active histone H1 kinase. This finding contrasts with the specificity of binding of Opisthokont cyclin B to the CDKA1 ortholog CDK1 [1]. As discussed in the Introduction, regulation of CDKB by cyclin B may have evolved early in Archaeplastida evolution.

### Cell-cycle-regulated and APC-dependent CYCB1 proteolysis

APC inactivation greatly increased CDKB1-associated kinase activity, without altering CDKB1 levels [29], and we inferred that this was likely due to blocking APC-dependent CYCB1 proteolysis. Here we show that CYCB1 abundance is indeed sharply cell-cycle-regulated. For a brief period surrounding cell division, the estimated half-life of CYCB1-GFP protein is reduced to ~3–5 min., and degradation is dependent on a functional APC. CYCB1-GFP degradation is sharply synchronous within daughter progeny in a single mother cell undergoing multiple fission. The basis for this synchrony (whether due to identical timing in independent progeny cells, or communication between cells) is unknown.

Cyclin B accumulation is required for mitotic entry in all eukaryotes examined; and cyclin B degradation is then essential for mitotic exit and entry into a new cell cycle in most but not all systems, meaning that cyclin B obligatorily cycles in abundance ([1]; see Introduction). Our results in *Chlamydomonas*, including inability to complement *cycb1-5* ts-lethality by transformation with *CYCB1-db*:*GFP-TG* (with the conserved destruction box deleted) is consistent with APC-mediated CYCB1 degradation being essential in *Chlamydomonas* as well. We speculated previously that CYCB1 might inhibit completion of cytokinesis, since elevating CYCB1 levels by APC inactivation is associated with absence of a cleavage furrow, while *apc;cycb1-5* double mutants form an aberrant partial furrow similar to that produced by *cycb1-5* single mutants [29].

The observation that CYCB1-GFP degradation is genetically dependent on CDKB1 as well as on the APC could be explained in two ways: (1) Degradation might be restricted to CYCB1 in a complex with CDKB1; (2) CYCB1-CDKB1 might be required to activate the APC. The former may be unlikely since in other organisms, APC-dependent degradation generally transfers with the destruction box, even if appended to reporters [8]. The latter mechanism could be consistent with results in animal cells, where APC-Cdc20 activation is dependent on cyclin B-Cdk1 phosphorylation of APC subunits [74]. The mechanism is complex: recruitment of Cks1-CDK-cyclin to a disordered region of APC3, promoting phosphorylation of a segment of APC1 that occludes the Cdc20 binding site; phosphorylated APC1 does not occlude the site and Cdc20 is recruited [74]. The regions and phospho-sites in human APC3 and APC1 identified as critical for this mechanism align poorly or not at all to the *Chlamydomonas* homologs, so any similar mechanism must be operating with divergent sequences. In tetrad analysis of crosses between *cdc20-1* and *cks1-1* [45], we observed complete lethality of the double mutants at permissive temperature, suggesting some collaboration between CDC20 and CKS1, but we have no information specifically connecting this to CDC20 activation by CDK.

### CDKB1 levels are regulated by entry into ‘division phase’ but not by cell cycle position

*CDKB* transcription and protein accumulation is elevated in mitotic cells in *Cyanodioschizon*, *Ostreococcus*, *Physcomitrella*, and *Arabidopsis* (red and green algae, moss, and land plant) [27,32]. This leads to the model that its degradation after mitosis could be cell-cycle-phase-specific, perhaps serving the same function as cyclin B degradation, to allow mitotic exit [32,37]. Our results show that in *Chlamydomonas*, this is not so. Unlike cyclin B, CDKB1 (the sole CDKB family member) is not removed at the conclusion of each mitosis. Rather, CDKB1 is restricted to what we call ‘division phase’: a condition of commitment to cell divisions (whether one or many) [30,31]. Cells undergoing multiple divisions make CDKB1 before the first division, and it stays high until all divisions are complete (and cells exit ‘division phase’); it is then rapidly degraded. The distinction between mitosis-specific accumulation and division-phase-specific accumulation is more easily made in *Chlamydomonas* as a result of multiple fission biology.

We recently suggested an equivalence between classical ‘commitment’ to division, and the activation of transcription of a large number of division-essential genes including *CDKB1* [30]. We speculate that transcription of these genes may be continuous throughout the period of multiple fission; lack of any drop in CDKB1 protein levels between divisions is consistent with this idea. We found recently that the replication control protein MCM4, a member of the mitotic transcriptional regulon along with CDKB1, accumulates as cells enter division phase, remains at a high level until the terminal division, then is degraded [68], thus exhibiting similar behavior to CDKB1. This is consistent with the idea of ‘division phase’ as a discrete cellular state, permissive for cell cycle progression but independent of specific cell cycle phase [30,31]. CDKB1 is not degraded in a *cdc27-6* background (Fig 3). This APC dependence of CDKB1 degradation is likely indirect, since CDKB1 lacks a recognizable target for APC-dependent degradation (D-box or KEN box). The APC is required for exit from division phase, and CDKB1 may remain stably accumulated at a high level for this reason.

### Regulation of microtubule structures by CYCB1-CDKB1 and APC-CDC20

EB1-NG localization, reflecting location of actively growing MT plus ends, was shown to be an informative single-cell marker for mitotic progression in *Chlamydomonas* [57]. As cells enter mitosis, EB1-NG localization undergoes dramatic changes [57]: the single polar focus of EB1-NG splits into two and separates; the mitotic spindle then forms between these two foci and persists for ~4 min before anaphase. Specifically during this period, the cortical comets characteristic of interphase cells [55] are entirely suppressed (Fig 11). After spindle breakdown, EB1-NG signal immediately moved to a ‘line’ perpendicular to the former spindle axis, marking the growing cleavage furrow. (Note that since this ‘line’ is detectable in a single plane of focus (Fig 7 and S4 Video), it likely reflects a surface of EB1-NG perpendicular to the former spindle axis, such as is created by the cleavage furrow [75]. Cleavage in *Chlamydomonas* is strongly dependent on microtubules [56], and can occur in the complete absence of F-actin [57], so the furrow localization of EB1-NG likely reflects essential microtubule growth during cytokinesis. We speculate that this line of EB1-NG reflects growth of the microtubule array called the ‘phycoplast’, which marks (and is probably required for) cleavage furrow development [56,57]. The four-membered rootlet microtubules run adjacent to the cytoplasmic microtubules in this array [53], and may dictate its location [53,56].

CYCB1/CDKB1 is required for the first step in this process; arrested *cycb1* cells keep a single anterior focus which does not split (Fig 8). Because no spindle forms in these cells, polar splitting in wild type may produce poles required for spindle generation. The mutant cells form an initial cellular indentation (the ‘notch’; [28]) (Fig 9), at the position of the anterior EB1-NG focus, but no extension of a line of EB1-NG into the cell (as observed in full cytokinesis; [57]; Fig 7) is observed.

Localization of CYCB1 to the region of the spindle poles 1–2 min before spindle formation is consistent with a direct regulation of the microtubule-organizing activity of spindle poles, though we cannot exclude an indirect mechanism at present. In animal cells and in fission yeast, cyclin B localizes to centrosomes via the cyclin B ‘hydrophobic patch’ docking motif ([76] and references therein); and *Chlamydomonas* CYCB1 retains all key residues making up the hydrophobic patch. However, in contrast to *Chlamydomonas*, cyclin B localization to spindle poles in yeast and animals may occur long before actual spindle formation. It is also important to note that in *Chlamydomonas*, the spindle pole is spatially distinct from the basal body (centrosome equivalent) [70]. We do not know whether CYCB1 localization is specific to one or the other of basal bodies or spindle poles.

Inactivation of APC or CDC20 has no effect on EB1 polar splitting or spindle formation, but anaphase, cleavage and cytokinesis are completely blocked; consistently, no ‘line’ of EB1-NG signal perpendicular to the spindle axis is observed in these blocked cells (Figs 9 and 10).

These results imply strong and opposing effects on formation and breakdown of microtubule structures by CYCB1/CDKB1 versus APC-CDC20. These events occur in stereotyped time intervals coordinated (and likely caused by) tight sequential changes in CYCB1 levels and APC-CDC20 activity.

*Chlamydomonas* has a gene orthologous to the *CDC20* homolog *CDH1*, which in other organisms can also activate the APC. The largely consistent results with EB1 comparing *cdc20-1* and *cdc27-6* mutants suggest that these phenotypes are due to loss of the APC^CDC20^ complex, and that *Chlamydomonas* CDH1 is unable to fully substitute for CDC20. This is also true in yeast and animals, owing at least in part to CDK-dependent inhibition of CDH1-APC [10]. The *Chlamydomonas* CDH1 homolog has multiple potential N-terminal CDK phosphorylation sites [77] which suggests the possibility of a similar regulatory mechanism.

The APC in *Chlamydomonas* very likely has a key role in regulating sister chromatid cohesion, independent of its role in regulating cyclin B levels; this regulatory structure is highly conserved [10]. The independent branch of APC activity leading to rounds of DNA replication is largely due, in budding yeast, to cyclin B-CDK-dependent inhibition of replication origin reloading. This in turn results in a requirement for APC inactivation, since the firing of those loaded origins requires cyclin B-CDK; thus, a complete cycle of cyclin B/APC activity is required for each round of replication [1]. In animals, the APC is responsible for degradation of geminin, an inhibitor of origin reloading [1]. Geminin is not found in yeast, and there is no clear homolog in the plant kingdom either [78]. How DNA replication is restricted to once-per-cell-cycle, and how the APC and/or cyclin-CDK activity might be involved, is not understood in the plant kingdom overall, though a connection to origin loading seems likely [68].

Regulation of microtubule dynamics and spindle morphogenesis by cyclin B-CDK may be conserved throughout eukaryotes [76,79] including the early-diverging plant kingdom ([51]; our results). Overall, we observe strong conservation between *Chlamydomonas* and yeast and animals of the roles of cyclin B/CDK and the APC with respect to their inter-regulation and their overall effects on cell cycle biology including spindle morphogenesis. This conservation strongly implies that these regulatory systems were already in place at the time of the last eukaryotic common ancestor [21]. However, while cyclin B has a highly conserved role in mitosis, its associated kinase subunit is CDKB1 rather than CDK1/CDKA1 as in yeast and animals; this substitution may be universal within the plant kingdom [27–29,32,51]. In the plant kingdom, CDKA1 may instead be specific to cell size control and the G1/S transition [27,30]. *Chlamydomonas* thus provides a unique opportunity to investigate the molecular regulation and mitotic functions of the plant-specific mitotic inducer CYCB/CDKB in a unicellular system, at high spatial and temporal resolution.

## Materials and methods

### Immunoprecipitation, immunoblotting, and kinase assay

Cell cultures used for immunoblotting of the time course of CYCB1-GFP in wt, *cdc27-6*, or *cdkb1-1* backgrounds (S1 Fig) were collected and equilibrated using optical density (OD) measured with a 750 nm light in a spectrophotometer. Cells were synchronized by nitrogen deprivation as follows. Equilibrated cell suspensions were put on TAP plates containing 1/10 the normal amount of ammonium chloride, and incubated for 24 hrs. at 21°C under light. Cells were then transferred to TAP plates with the normal amount of ammonium chloride and moved to 33°C under light. When transferred to 33°C, cell suspensions from each strain were split equally to separate TAP plates (250 mL each). At each collection time point, the entire cell contents of one of these replicate plates were collected for immunoblotting. Western blotting was carried out using standard methods. The anti-GFP antibody (GF28R, Thermo Scientific) was used at 1:2000 dilution. Signal was detected by chemiluminescence (SuperSignal West Femto, Thermo Scientific) in an ImageQuant LAS 4000 imager (GE Healthcare).

Cells used for the CYCB1-GFP immunoprecipitation Western blot and kinase assay (Fig 1A) were synchronized by nitrogen deprivation as described above, then placed at 33°C, and collected after 13 hrs. at 33°C. Two replicates were collected for each genotype. The same biomass was collected from all cultures, equalized based on OD. The collected cultures had the following OD values: untagged WT #1, 0.70; untagged WT #2, 0.45; WT #1, 0.55; WT #2, 0.70; cdc27-6 #1, 0.52; cdc27-6 #2, 0.62; cdkb1-1 #1, 0.59; cdkb1-1 #2, 0.45. The same volume (5 mL) was spun down from all cultures, then they were resuspended in a number of milliliters equaling the OD values above. Finally, 0.2 mL were collected from all of the resuspended cultures.

Immunoprecipitation was carried out as described in [29], except that CYCB1-GFP was pulled down with the anti-GFP nanobody LaG41, which was a gift from Peter Fridy and Michael P. Rout [80]. A small sample of the total cell lysates was used for Western blotting for CYCB1-GFP detection, then immunoprecipitation with the anti-GFP nanobody was done on the remaining total cell lysates. All Western blotting was done with standard methods using anti-GFP antibody GF28R (Thermo Scientific) at 1:2000 dilution. The kinase assay was done as previously described [29].

In the experiment for the detection of CDKA1-mCherry or CDKB1-mCherry by immunoprecipitation of CYCB1-GFP (Fig 1B), cells were synchronized by nitrogen deprivation and collected after 13 hrs. at 33°C, as described above. The collected cultures had the following OD values: WT #1, 0.44; WT #2, 0.49; CYCB1-GFP #1, 0.33; CYCB1-GFP #2, 0.51; CDKA1-mCherry #1, 0.50; CDKA1-mCherry #2, 0.63; CYCB1-GFP CDKA1-mCherry #1, 0.59; CYCB1-GFP CDKA1-mCherry #2, 0.78; CDKB1-mCherry #1, 0.62; CDKB1-mCherry #2, 0.88; CYCB1-GFP CDKB1-mCherry #1, 0.52; CYCB1-GFP CDKB1-mCherry #2, 0.88. The biomass collected was equalized in the same way as in the experiment in Fig 1A, described above.

In this experiment (Fig 1B), immunoprecipitation of CYCB1-GFP was done with the same method described above and with the same anti-GFP nanobody (LaG41). Western blotting of the co-immunoprecipitated CYCB1-GFP and CDKA1-mCherry or CDKB1-mCherry was done with standard methods as described above. The anti-GFP antibody (GF28R, Thermo Scientific) was used at 1:2000 dilution. The anti-mCherry antibody (16D7, Thermo Scientific) was also used at 1:2000 dilution.

### Strain construction and growth

After initial transformation with transgenes, all strain constructions were by mating and tetrad analysis. *cdkb1-1* and *cdc27-6* (there called *div23*) were isolated in [28] and characterized in [28,29]. *cycb1-5* was characterized in [29]. *cdc20-1* was isolated and characterized in [45]. *scc2-3* (chr6:C4382319T; G600E; was isolated following the procedures in [45].

Temperature-sensitive mutations and trans-genes were multiply backcrossed to wild type (in most cases 4–5 times) before analysis, and multiple independent strains tested, to ensure that results were due to the indicated genotypes and not to unknown background mutations. Strains were maintained in continuous light in TAP medium.

### Fluorescent reporter constructs

#### CYCB1:GFP-TG

We constructed a plasmid with 1.3 kb of genomic DNA upstream of *CYCB1*, followed by the *CYCB1* coding sequence with introns; the termination codon was replaced with 3 copies of a GlyGlyGlyGlySer linker sequence followed by GFP. After the GFP termination codon the plasmid contained 1.1 kb of the 3’ UT region from *CDKB1*, followed by a 1 kb fragment containing a paromomycin resistance cassette.

We linearized this plasmid and transformed a *cycb1-5* strain by electroporation as described [29]. We recovered transformants in two ways: either by selection on paromomycin at 21 degrees (permissive temperature for *cycb1-5*) or by selection without paromomycin at 33 degrees (non-permissive temperature). For unknown reasons, likely related to the known fragmentation of transforming DNA in *Chlamydomonas*, all of the paromomycin-resistant colonies tested were temperature-sensitive, and none of the temperature-resistant colonies were paromomycin-resistant. We chose one temperature-resistant transformant and found linkage in tetrad analysis between a single locus containing *GFP* by PCR, and rescue of temperature-sensitivity of *cycb1-5*. Parallel transformations with an identical plasmid with a deletion of the *CYCB1* destruction box [29] failed to yield any temperature-resistant transformants in three experiments, while *CYCB1-GFP* with an intact destruction box yielded more colonies than could be reliably counted but at least 100 in each experiment. Generally low linkage of paromomycin resistance to *CYCB1-GFP* (presumably due to frequent degradation of transforming DNA in *Chlamydomonas*) precluded the use of this marker for further analysis of the lack of rescue by *CYCB1-db-GFP*.

*Chlamydomonas* transgenes are frequently subject to random silencing [81]. We largely eliminated this problem with *CYCB1*:*GFP-TG* by keeping *cycb1-5* in the transgenic strain background, and selecting cultures at non-permissive temperature before time-lapse microscopy (see below). Even with this precaution, we observed sporadic cells in time-lapse that failed to express *CYCB1*:*GFP-TG*, instead arresting with the characteristic morphology of *cycb1-5* [29].

#### EB1

To construct pMO699 (EB1-mSC), the mNeonGreen sequence in pCrEB1-NG [55] was excised out using *Xho*I sites and replaced with mScarlet-I [amplified from mScarlet-I-mTurquoise2 (Addgene, Plasmid #98839) [82] by Gibson assembly. pMO669 was then linearized using *Eco*RI and *Sca*I prior to transformation into *Chlamydomonas* by electroporation.

In one experiment (Fig 12 and S9 Video) we used an allele of *CYCB1*:*GFP* in which the endogenous copy of *CYCB1* was tagged with GFP. The endogenously tagged CYCB1-GFP behaved similarly to the transgene-expressed CYCB1-GFP used in all other experiments reported here. All strains expressing endogenously tagged EB1-mNG or CYCB1-GFP are progeny of single parental strains made by CRISPR-mediated tagging. The CRISPR procedure was done largely as previously described [83] using Alt-R sgRNA and S.p.Cas9 Nuclease V3 from Integrated DNA Technologies. Sequence of sgRNA used for EB1 tagging: GAAACACAAGTGGAGTTCGCGGG. Sequence of sgRNA used for CYCB1 tagging: ACAGGGAAAGCACAGCTCATGGG.

#### Time-lapse Microscopy

Multiple imaging methods were used. Method #1 was used for single Z-plane imaging at 3 min. intervals and low fluorescence exposure times to avoid cell phototoxicity and to image cells through multiple division cycles. Method #2 was used for 10- or 20-second interval movies at a single Z-plane. Method #3 was used for 1-minute interval movies with high fluorescence exposure times. These methods are complementary; frequent exposures, high exposure times and multiple Z planes allowed high-resolution detection of events within a single cell division, but the imaged cells generally lost viability soon after; while the much lower overall illumination of Method #1 reduced temporal and spatial resolution but allowed reliable imaging of an entire multiple fission cycle (at the end of which viable cells frequently hatched from the mother and swam away, demonstrating a complete successful multiple fission cycle).

#### Method #1

Cells were taken from a 2-day culture on a TAP plate, transferred to liquid TAP for 4 hrs. for cells to become motile, then swimming cells were separated from dividing and other non-motile cells and debris. This separation was achieved by pipetting 500 μL of the liquid cell culture, removing the pipette tip from the pipette, then placing the pipette tip into another tip containing 500 μL TAP + 2% Ficoll, such that the end of the pipette tip containing the cells was in contact with the Ficoll. A white LED (Evan Designs) was then placed on the wide end of the pipette tip pair. This complete apparatus was then put in a dark enclosure. With the LED being the only source of light inside the enclosure, cells swim away from the light, into the Ficoll, and collect on the end of the pipette tip. Once a sufficient number of motile cells had traversed the Ficoll and accumulated on the pipette tip, the pipette tip was removed, and the cells were pushed out by lightly pressing the wide end of the pipette tip. This ‘swim-selected’ population mostly consisted of small to medium motile cells (due to the high density of the Ficoll), with very few large or dividing cells carried over by the flow of the smaller cells.

To immobilize the swim-selected cells for long-term time-lapse microscopy, they were placed on agarose medium immediately after collection, similar to what was used by Di Talia et. al. [84] for budding yeast microscopy. However, the setup employed by Di Talia et. al. [84] involved large agarose slabs placed close together on a glass cover slip, and covered with a clear plastic piece, which was then sealed along the edges with paraffin. This setup could not be used for long-term microscopy of *Chlamydomonas* for the following reasons. First, unlike budding yeast, *Chlamydomonas* cells are motile, so placing agarose slabs close together on a cover slip allows for the possibility of cells swimming from one slab to the other (if a connective layer of liquid is formed between the slabs). Second, in order to make long-term movies (20 hrs.), drying of the agarose must be minimized. A large plastic cover placed over the agarose slabs results in enough drying during the course of the movie that cells often drift completely out of the field. Drying causes cells to move along the z-axis as well, necessitating a very large autofocus range. A large plastic cover also collects water on its inner surface by condensation, which was significant at the temperature at which we intended to make movies (33.3°C).

To avoid these issues, we designed a small cylindrical chamber with one open side (inner diameter: 5mm; inner height: 3.5 mm; wall diameter: 1 mm). The chamber was fabricated from clear acrylic sheets using a laser cutter at Rockefeller University’s Precision Instrumentation Technologies facility. The barrel portion of the chamber was made by cutting two concentric circles on a 3.5 mm-thick acrylic sheet. The inner circle had a diameter 2 mm smaller than the outer circle, so that the sides of the barrel were 1 mm wide. A lid was made by cutting a clear 1 mm-thick acrylic sheet in a circle with a diameter equal to the outer diameter of the barrel. The lid was then attached to the barrel with acrylic cement (Scigrip).

Molten TAP with 1.5% SeaKem NuSieve GTG agarose (Lonza) was poured into the chamber to a level of 3 mm above the top and a glass slide was placed 1.5 mm above the upper edge of the box, flattening the agarose. The agarose was allowed to solidify at room temperature for 10 min., then the glass slide was removed. Cells were pipetted (0.5 μL) onto the agarose surface and kept at room temperature for 15 min. to allow the surface to dry. The agarose edges were trimmed so that the exposed agarose surface was flat throughout. The cell side of the box was placed onto a 24 x 50 mm glass cover slip (Fisherbrand) and the exposed agarose portion was sealed with VALAP (equal mass petroleum jelly, paraffin, lanolin). When multiple cell chambers were used, they were placed 1 cm apart (center-to-center). Plastic cover slips (Rinzle and ACLAR, both from Electron Microscopy Sciences) occasionally resulted in better cell viability and division number compared to glass (mostly 3 divisions compared to 2 divisions on glass), but this difference was irregular between batches. Glass cover slips from our current supplier (Fisher Scientific ‘Fisherbrand’ Cat. No. 12-545-F), have been consistently better than Rinzle or ACLAR plastic in maintaining cell viability and most cells divide 3–4 times. Glass has the additional benefits of lower autofluorescence compared to plastic, and less flexion, which results in less drift along the z-axis, making autofocusing easier.

Time-lapse microscopy was carried out on a Leica DMI6000B inverted microscope, using a 63X objective, with the objective and stage heated to 33.3°C. Images were acquired using custom software, as previously described for budding yeast microscopy [85], but with modifications to improve autofocus for *Chlamydomonas*. We acquired brightfield images instead of phase contrast, because brightfield allowed for more reliable autofocusing. Fluorescence images were acquired using a Leica EL6000 mercury-arc lamp and a 30% neutral density filter. GFP images were acquired with 0.4 s exposure using a narrow-band eGFP filter set from Chroma (Cat. No. 49020) to minimize autofluorescence. Venus and mNeonGreen (NG) images were acquired with 0.3 s exposure using an eYFP filter set from Chroma (Cat. No. 49003). For chloroplast background, we acquired images with 0.003 s exposure using a Cy5 filter set from Chroma (Cat. No. 49006).

In *Chlamydomonas*, chloroplasts fluorescence is detectable at most wavelengths, and this seriously interferes with detection of the rather dim CYCB1-GFP signal. We developed a simple deconvolution procedure to subtract chloroplast background from GFP (see below).

To provide the cells with illumination for photosynthesis between frames, we placed white LEDs (Evan Designs) 10 mm above the cell chambers and 7 mm away from the imaging axis, so that the irradiance at the location of the cells was 150 μmol photons m^-2^ s^-1^. The LEDs were mounted on a 3D-printed plastic enclosure that covered the cell chamber. The transmitted light path from above was not impeded because a clear plastic ACLAR film (Electron Microscopy Sciences) was used as a top. This enclosure also helped maintain temperature stability of the cell chamber by partially insulating against ambient temperature fluctuations. The LEDs were connected to a computer-controlled on/off timer (PowerUSB). The LED lights were off for the duration of the transmitted light/fluorescence image acquisition, then on for most of the remaining time until the subsequent frame. Because of imperfect synchrony between the time-lapse image acquisition schedule and the exterior LED light on/off timer, 10–20 sec. were added to the LED off time, allowing a minimum of 90 sec. of LED illumination between 3-min. frames.

Temperature stability and accuracy during the course of a time-lapse movie was extremely important. We found that in the microscopy setup described above, wild-type cells are inviable at 34°C and above. Many temperature-sensitive mutants do not arrest tightly below 33°C. Therefore, our movies were done at 33.3°C (± 0.3°C). To measure the temperature exactly at the location of the cells, we embedded a 0.1 mm diameter thermocouple (PerfectPrime TL0201) in the agarose microscopy chamber. To maintain this small temperature range, we heated the objective (with an aluminum collar) and stage (with an aluminum insert) with Peltier modules run by Oven Industries 5C7-195 controllers. To minimize the effect of air currents above and below the stage, we covered openings below the stage with aluminum foil, and used a 3D-printed plastic enclosure above the stage. The enclosure was printed at Rockefeller University’s Precision Instrumentation Technologies facility.

#### Method #2

As described previously [57].

#### Method #3

Cells were synchronized using the 12L:12D light cycle at 26°C. At ~11 h, the cells were collected by centrifugation and spotted on a small block of TAP + 1.5% low-melting-point agarose (Bio-Rad), which was then placed in a glass-bottomed 18-well chamber (Ibidi) and sealed with additional TAP + low-melting-point agarose. Imaging was done using a Leica Thunder inverted microscope equipped with an HC PL APO 63X/1.40 N.A. oil-immersion objective lens and an OkoLab incubator chamber that was maintained at 27°C. Signals were captured using following combinations of LED excitation and emission filters: 510 nm and 535/15 nm for CYCB1-GFP and EB1-NG; 550 nm and 595/40 nm for EB1-mSc; 640 nm and 705/72 nm for chlorophyll autofluorescence (AF). Time-lapse images were captured at 2-min intervals with 0.6 μm Z-spacing covering 9 μm; still images were captured with 0.21 μm Z-spacing covering 10–15 μm. The acquired fluorescence images were processed through Thunder Large Volume Computational Clearing and Deconvolution (Leica). Background chloroplast signal was removed from GFP images essentially as described below. Maximum projections from 15 z-stacked images of CYCB1-GFP and EB1-mSC were used in Fig 12 and S9 Video. CYCB1-GFP maximum projections were grainy because the signal was close to background; this problem was reduced by a Gaussian blurring of the GFP stack before the maximum projection was calculated (0.5 * image (n-1) + image(n) + 0.5 * image(n)).

### Quantification of EB1-NG signals

The “Peak” mask representing the polar dots, mitotic spindle, and furrow, was created from MAX-projected EB1-NG images by applying Gaussian blur filtering (1.5 pixels) and a Default thresholding filter in ImageJ. A mask representing the total cell body was generated from MAX-projected AF images by applying Gaussian blur filtering (2 pixels) and a Triangle thresholding filter. Subtraction of the “Peak” region from this mask yielded a mask essentially covering the cytoplasm. Unlike the mid-section images shown in the figures, this MAX projection covers most of the cell body after binarization using an appropriate threshold, except that the thin cortical layer is not covered. Strong 2-pixel blur was applied to expand the signal so that the resulting mask covers the cortex. In interphase cells, EB1 signal under this cytoplasmic mask was mainly due to discrete ‘comets’ of EB1 traveling along the cortex to the cell posterior [55]. Signals of EB1-NG were quantitated in each mask after uniform subtraction of background corresponding to intensities in non-cell areas.

### Deconvolution for time lapse image analysis

Autofluorescence from chloroplasts accounted for a large majority of the total signal with CYCB1-GFP or CDKB1-Venus detection. We developed a simple computational deconvolution procedure that largely corrected this problem. The key observation is that due to the broad excitation and emission spectra of photosynthetic pigments, chloroplasts are detectable with filters specific for GFP, YFP or RFP; in contrast, GFP and YFP have no signal under RFP detection. The brightest RFP signal was invariably detected in the posterior region of the cell where chloroplasts are known to reside. Therefore, assuming that chloroplast pigments have the same ratio of GFP:RFP detection at all points in the cell, it is straightforward, given paired images for GFP and RFP detection, to determine this ratio from high-RFP pixels (presumably deriving purely from chloroplast), and then to deconvolve the GFP-specific signal throughout the image (S2 Fig). This deconvolution is carried out automatically using the same algorithm for every image. To account for possible variations in lamp intensity or exposure time through a movie, the deconvolution ratio is calculated separately for each image in the series. Suppose F is the average ratio of red to green signal in the pixels with the highest red signal (pure chloroplast). Consider another pixel potentially containing both chloroplast and CYCB1-GFP signal. If CYC is the amount of CYCB1-GFP contributing to signal from that pixel, and the total green and red signals from that pixel are G and R respectively, then

G = FR + k*CYC, where k is a constant reflecting green emission from a given amount of CYCB1-GFP. Therefore, amount of CYCB1 in that pixel (in arbitrary units) is:

CYC = (G-FR)/k

Assuming similar lamp intensity and exposure through the movie, k is a constant that is buried in arbitrary units for CYCB1-GFP. Given the assumptions above, this results in a linear measure of CYCB1-GFP comparable across an image and between images in a series, with the contribution of chloroplast to green signal removed.

This method does depend on a sufficient true GFP signal to overcome minor fluctuations in chloroplast intensity, chloroplast movement between exposures, etc. We think that S2 Fig justifies the system in the present case; clearly, the major effect of the deconvolution is to drop out background not due to GFP, and the procedure does not introduce artefactual peaks or valleys that are not present in the initial un-deconvolved data.

The same procedure works for YFP.

MATLAB code to carry out the deconvolution is available on request.

## Supporting information

S1 Fig Detection of CYCB1-GFP in wild-type, *cdc27-6*, or *cdkb1-1* backgrounds by immunoblotting. Anti-GFP immunoblotting of CYCB1-GFP in wild-type, *cdc27-6*, or *cdkb1-1* backgrounds. Cells were blocked in G1 by nitrogen deprivation, as described [29], then placed at restrictive temperature (33°C) in complete medium, and collected after the indicated number of hours. All strains had temperature- sensitive *cycb1-5* rescued by *CYCB1*:*GFP-TG* transgene. Loading control is from non-specific anti-GFP signal from immunoblot. CYCB1-GFP detection was from the same exposures for WT and mutants assayed in parallel; loading control (non-specific band reacting with anti-GFP antibody) was from a different exposure but similarly comparable between strains.
(TIFF)

S2 Fig Illustration of subtraction of chloroplast autofluorescence in microscopy images. Autofluorescence subtraction method demonstrated using a single *CYCB1*:*GFP-TG* cell. First column: RFP detection (colored blue) (chloroplast signal only). Second column: GFP detection channel only (CYCB1-GFP signal + chloroplast signal). Third column: composite of first two columns. Fourth column: GFP signal remaining after deconvolution (removal of contribution of chloroplast signal to GFP channel, leaving CYCB1-GFP signal only). Fifth column: composite of RFP channel (chloroplast only) and deconvoluted green channel (CYCB1-GFP signal only). Bottom: quantification of total RFP and GFP signals (left), and GFP signal concentration (black line) before and after deconvolution.
(TIFF)

S3 Fig Live cell time-lapse of CYCB1-GFP in a *cdkb1-1* background. Live cell time-lapse acquired with microscopy method 1 (see Methods section). Each cell has a brightfield image (right), and a composite of CYCB1-GFP signal in yellow and chloroplast autofluorescence signal in blue (left). Time indicated on top of each strip is hours and minutes after beginning of time-lapse. The indicated time corresponds to the top cell in each strip. Each subsequent cell going down is from an image captured every 3 minutes. We assume the absence of any CYCB1-GFP signal in one frame at 11:21 is due to failure of excitation or shutter opening for the acquisition of this single frame; this is a rare, intermittent issue with the time-lapse setup used.
(TIFF)

S4 Fig Flow cytometry of wild-type, *cdc20-1, esp1-2* with or without *scc2-3*. (A) Wild-type, *cdc20-1*, *esp1-2* mutants with or without *scc2-3* were blocked in G1 by nitrogen deprivation, as described [29], then placed at restrictive temperature (33°C) in complete medium, collected after 13 and 15 hrs., and analyzed by flow cytometry. For each time point and genotype, the DNA content histogram (count vs. fluorescence signal) shows DNA complement number, and the forward scatter vs. fluorescence signal plot reflects cell size. See Tulin & Cross 2014 [28], Cross 2020 [30] for discussion on interpretation of the DNA and cell size signals in this procedure. The causative mutation in *esp1-2* was W2316R [28]. In separate experiments we compared the arrest phenotype of *esp1-2* to other alleles: *esp1-1* (W2211L), *esp1-4* (F2185L), *esp1-6* (L1284P) and *esp1-7* (W179R). All yielded high-ploidy cells by FACS (extending to 8C); microscopy of the FACS samples showed that almost all DNA staining was in a single large nucleus, as in Fig 4.
(TIFF)

S5 Fig Analysis of *cdc20;esp1* and *scc2;esp1* double mutants. (A) Stationary-phase cells from one tetrad from a *cdc20* x *esp1* cross were plated on fresh complete medium at restrictive temperature (33°C) and observed by microscopy at 13, 14, 15 hrs. The *cdc20;ESP1* and *cdc20;esp1* segregants were indistinguishable morphologically. (B) Comparison of segregants from an *scc2* x *esp1* cross patched on TAP-agar at permissive temperature (21°C), partially restrictive temperature (30°C), and fully restrictive temperature (33°C*)*. *scc2* mutations isolated in our screen [45] appear somewhat leaky, allowing limited proliferation at non-permissive temperature; the cells produced at this temperature are largely inviable [45].
(TIFF)

S6 Fig Flow cytometry of wild-type, *cdc20-1, esp1-2*, and *cdc20-1;esp1-2*. (A) Wild-type, *cdc20-1*, *esp1-2*, and *cdc20-1;esp1-2* cells were blocked in G1 by nitrogen deprivation, as described [29], then placed at restrictive temperature (33 C) in complete medium, collected after 13 and 15 hrs., and analyzed by flow cytometry. For each time point and genotype, the DNA content histogram (count vs. fluorescence signal) shows DNA complement number, and the forward scatter vs. fluorescence signal plot reflects cell size. As with cytokinesis, *cdc20* is completely epistatic to *esp1*.
(TIFF)

S7 Fig Traces for individual cells used for quantification in Fig 12. Top and bottom rows show the total signal under the green and magenta masks in Fig 12, respectively.
(TIFF)

S1 Video CYCB1-GFP through 3 divisions. Top left: blue line: RFP (chloroplast) signal. Yellow line: deconvolved **CYCB1-GFP** signal. Top 2^nd^ graph: yellow: total GFP signal; black: a minimal convex hull was calculated containing 50% of the total cell GFP signal and concentration calculated; 3^rd^: histogram of intensities in the convex hull; 4^th^: surface plot of GFP intensity. Below: brightfield (left) and fluorescence (right). Black line: manually selected cell outline. White line: computed convex hull.
(MP4)

S2 Video CYCB1-GFP in *cdkb1-1* background. Graphs and images as in S1 Video.
(MP4)

S3 Video Live cell time-lapse of first division in wild-type *EB1:NG-TG* cells, 10-sec. intervals. Live cell time-lapse with 10-sec. intervals acquired with microscopy method 2 (see Methods section). EB1-NG signal in blue.
(MP4)

S4 Video Live cell time-lapse of wild-type *EB1:NG-TG* cells carrying out 3 rounds of multiple fission, 3-min. intervals. Live cell time-lapse with 3-min. intervals acquired with microscopy method 1 (see Methods section). Image on left is brightfield, image on right is EB1-NG signal in yellow and chloroplast autofluorescence signal in blue.
(MP4)

S5 Video Live cell time-lapse of *esp1;EB1:NG-TG* cells. Live cell time-lapse acquired with microscopy method 1 (see Methods section), 3 min intervals. Image on left is brightfield, image on right is EB1-NG signal in green and chloroplast autofluorescence signal in red.
(MP4)

S6 Video Live cell time-lapse of *cycb1-5;EB1:NG* cells. Live cell time-lapse acquired with microscopy method 1 (see Methods section), 3 min intervals. Image on left is brightfield, image on right is EB1-NG signal in yellow and chloroplast autofluorescence signal in blue.
(MP4)

S7 Video Live cell time-lapse of *cdc27-6;EB1:NG-TG* cells. Live cell time-lapse acquired with microscopy method 1 (see Methods section), 3 min intervals. Image on left is brightfield, image on right is EB1-NG signal in yellow and chloroplast autofluorescence signal in blue.
(MP4)

S8 Video Live cell time-lapse of *cdc20-1;EB1:NG-TG* cells. Live cell time-lapse acquired with microscopy method 1 (see Methods section), 3 min intervals. Image on left is brightfield, image on right is EB1-NG signal in yellow and chloroplast autofluorescence signal in blue.
(MP4)

S9 Video Time-lapse microscopy of *CYCB1:GFP;EB1-mScarlet* cells. Live cell time-lapse of *CYCB1*:*GFP;EB1*:*mScarlet* cells with 1-min. intervals acquired with microscopy method 3, with 15 z-stacks (see Methods section). Left to right: DIC (time in min:sec indicated); EB1-mSC; CYCB1-GFP; chloroplast autofluorescence; overlap of CYCB1-GFP (green) and EB1-mSC (magenta). GFP images were deconvolved to remove chloroplast contribution (Methods). The GFP z images were filtered (0.5 * image (n-1) + image (n) + 0.5* (n-2) and then a maximum projection calculated. This procedure was developed for maximum detail while minimizing graininess. EB1-mSC images are a maximum projection. EB1-mSC cells lacking GFP show no bleedthrough from mSC to the GFP channel.
(MP4)
