# Supplementary figures and images for: Control of division in Chlamydomonas by cyclin B/CDKB1 and the anaphase-promoting complex
Source: PLoS Genet. 2022 Aug 18;18(8):e1009997. doi: 10.1371/journal.pgen.1009997 (PMC9448001; doi:10.1371/journal.pgen.1009997)

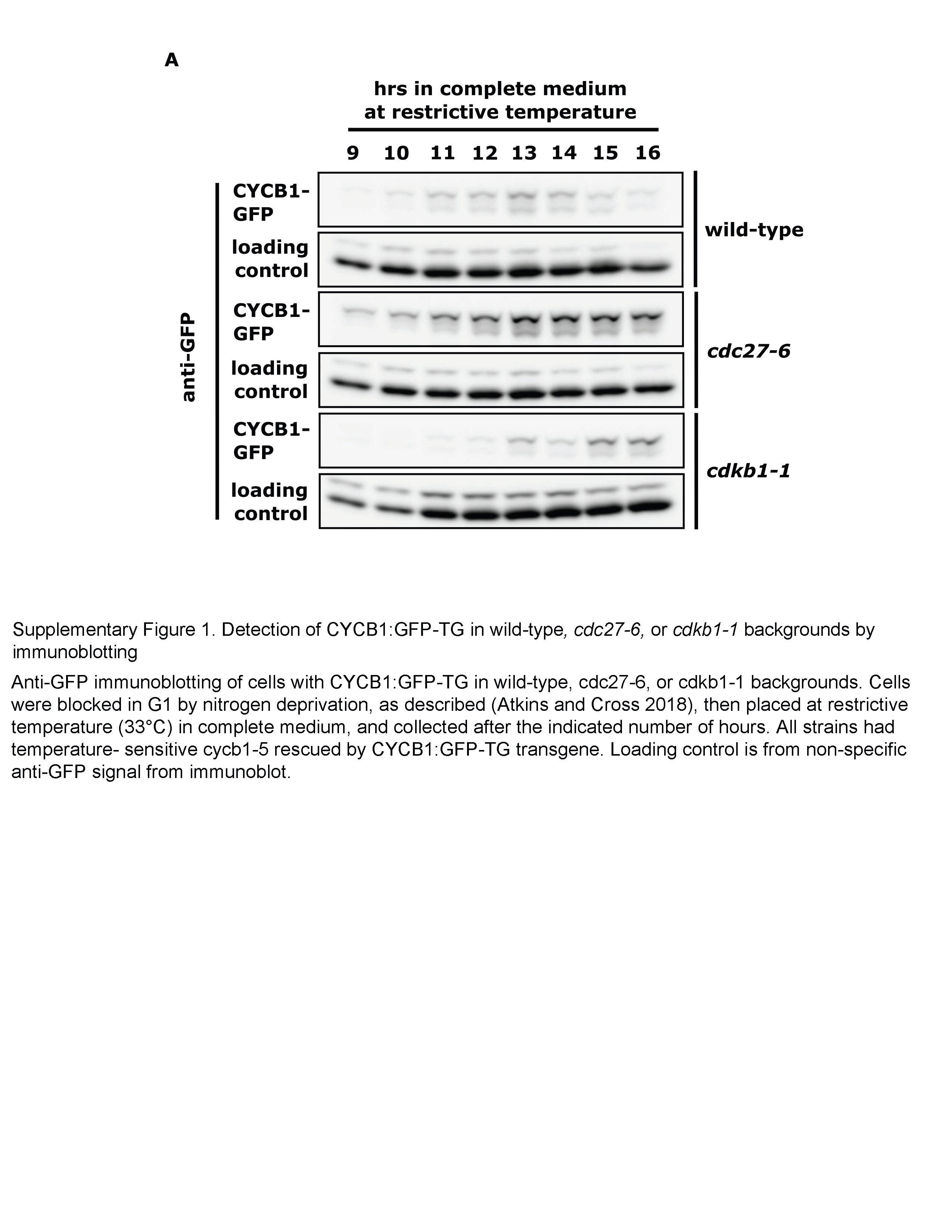

Supplement: S1 Fig — Anti-GFP immunoblotting of CYCB1-GFP in wild-type, cdc27-6, or cdkb1-1 backgrounds. Cells were blocked in G1 by nitrogen deprivation, as described [29], then placed at restrictive temperature (33°C) in complete medium, and collected after the indicated number of hours. All strains had temperature- sensitive cycb1-5 rescued by CYCB1:GFP-TG transgene. Loading control is from non-specific anti-GFP signal from immunoblot. CYCB1-GFP detection was from the same exposures for WT and mutants assayed in parallel; loading control (non-specific band reacting with anti-GFP antibody) was from a different exposure but similarly comparable between strains. (TIFF) [file pgen.1009997.s001.tiff]

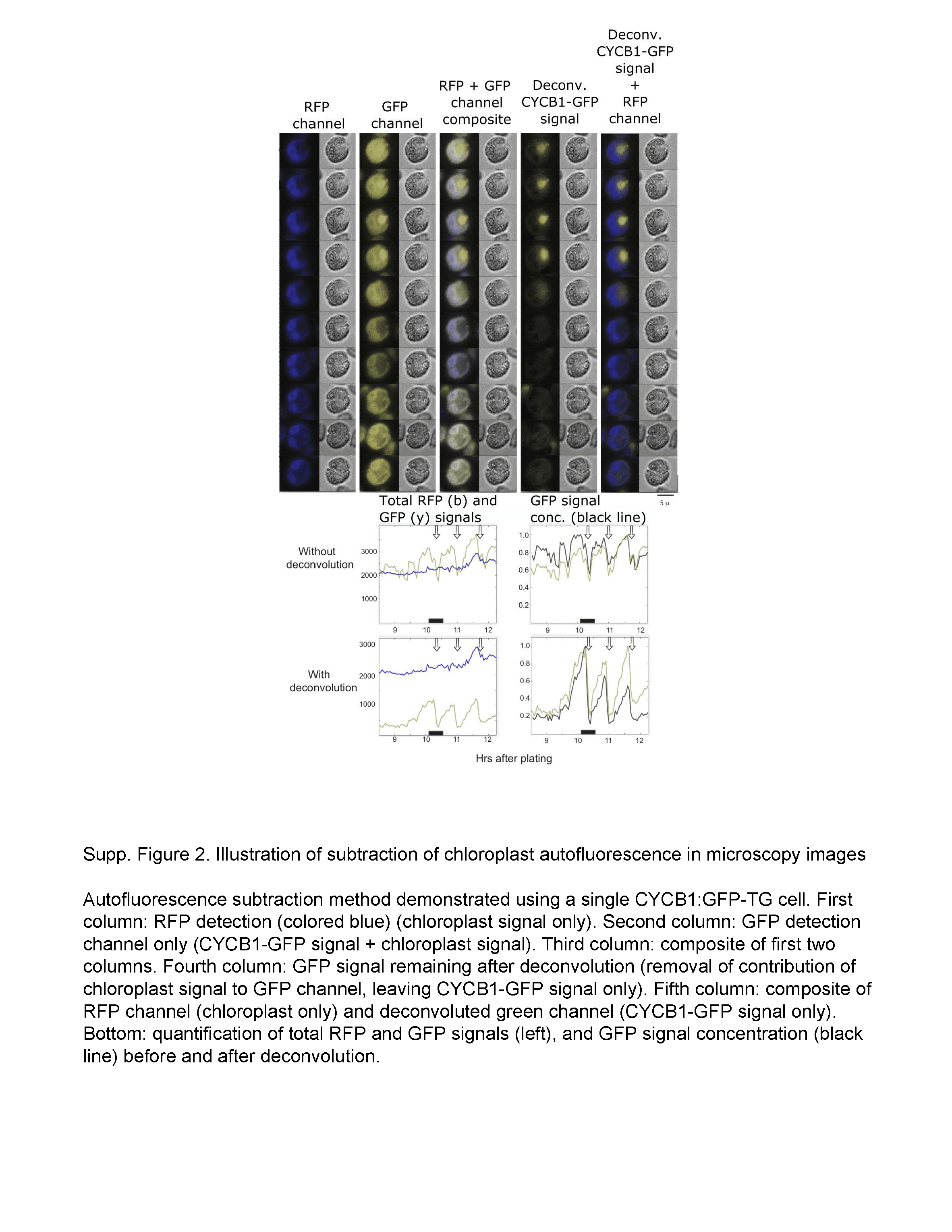

Supplement: S2 Fig — Autofluorescence subtraction method demonstrated using a single CYCB1:GFP-TG cell. First column: RFP detection (colored blue) (chloroplast signal only). Second column: GFP detection channel only (CYCB1-GFP signal + chloroplast signal). Third column: composite of first two columns. Fourth column: GFP signal remaining after deconvolution (removal of contribution of chloroplast signal to GFP channel, leaving CYCB1-GFP signal only). Fifth column: composite of RFP channel (chloroplast only) and deconvoluted green channel (CYCB1-GFP signal only). Bottom: quantification of total RFP and GFP signals (left), and GFP signal concentration (black line) before and after deconvolution. (TIFF) [file pgen.1009997.s002.tiff]

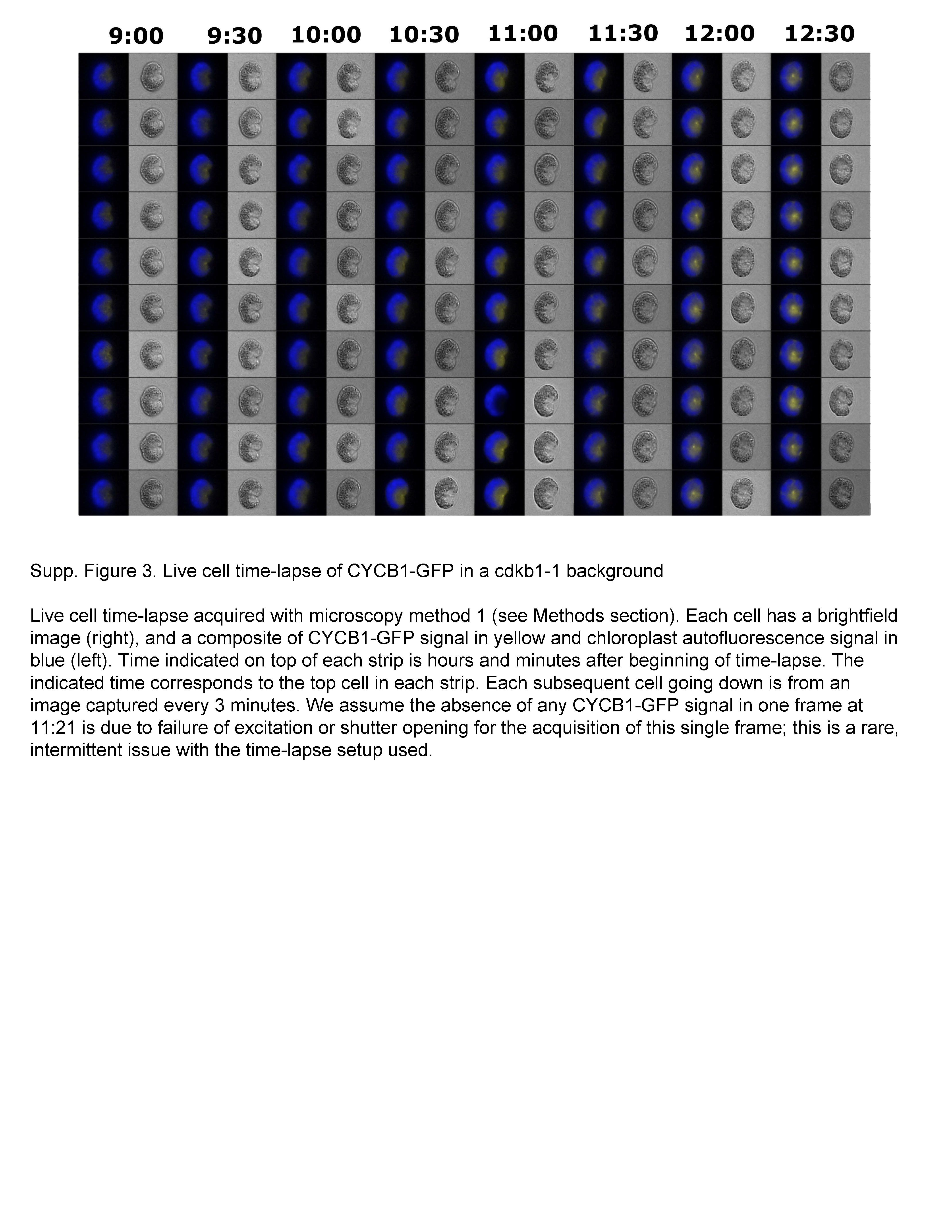

Supplement: S3 Fig — Live cell time-lapse acquired with microscopy method 1 (see Methods section). Each cell has a brightfield image (right), and a composite of CYCB1-GFP signal in yellow and chloroplast autofluorescence signal in blue (left). Time indicated on top of each strip is hours and minutes after beginning of time-lapse. The indicated time corresponds to the top cell in each strip. Each subsequent cell going down is from an image captured every 3 minutes. We assume the absence of any CYCB1-GFP signal in one frame at 11:21 is due to failure of excitation or shutter opening for the acquisition of this single frame; this is a rare, intermittent issue with the time-lapse setup used. (TIFF) [file pgen.1009997.s003.tiff]

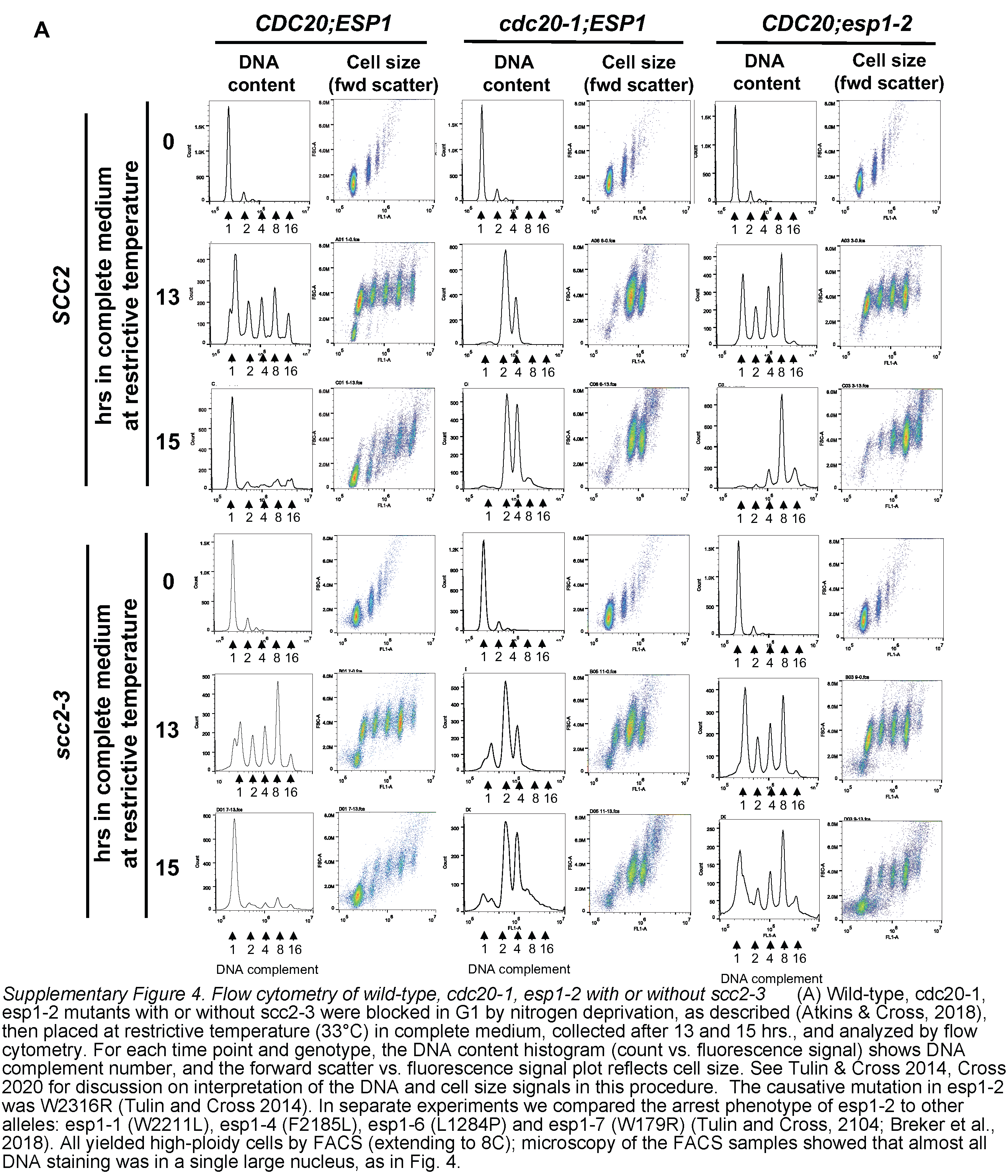

Supplement: S4 Fig — (A) Wild-type, cdc20-1, esp1-2 mutants with or without scc2-3 were blocked in G1 by nitrogen deprivation, as described [29], then placed at restrictive temperature (33°C) in complete medium, collected after 13 and 15 hrs., and analyzed by flow cytometry. For each time point and genotype, the DNA content histogram (count vs. fluorescence signal) shows DNA complement number, and the forward scatter vs. fluorescence signal plot reflects cell size. See Tulin & Cross 2014 [28], Cross 2020 [30] for discussion on interpretation of the DNA and cell size signals in this procedure. The causative mutation in esp1-2 was W2316R [28]. In separate experiments we compared the arrest phenotype of esp1-2 to other alleles: esp1-1 (W2211L), esp1-4 (F2185L), esp1-6 (L1284P) and esp1-7 (W179R). All yielded high-ploidy cells by FACS (extending to 8C); microscopy of the FACS samples showed that almost all DNA staining was in a single large nucleus, as in Fig 4. (TIFF) [file pgen.1009997.s004.tiff]

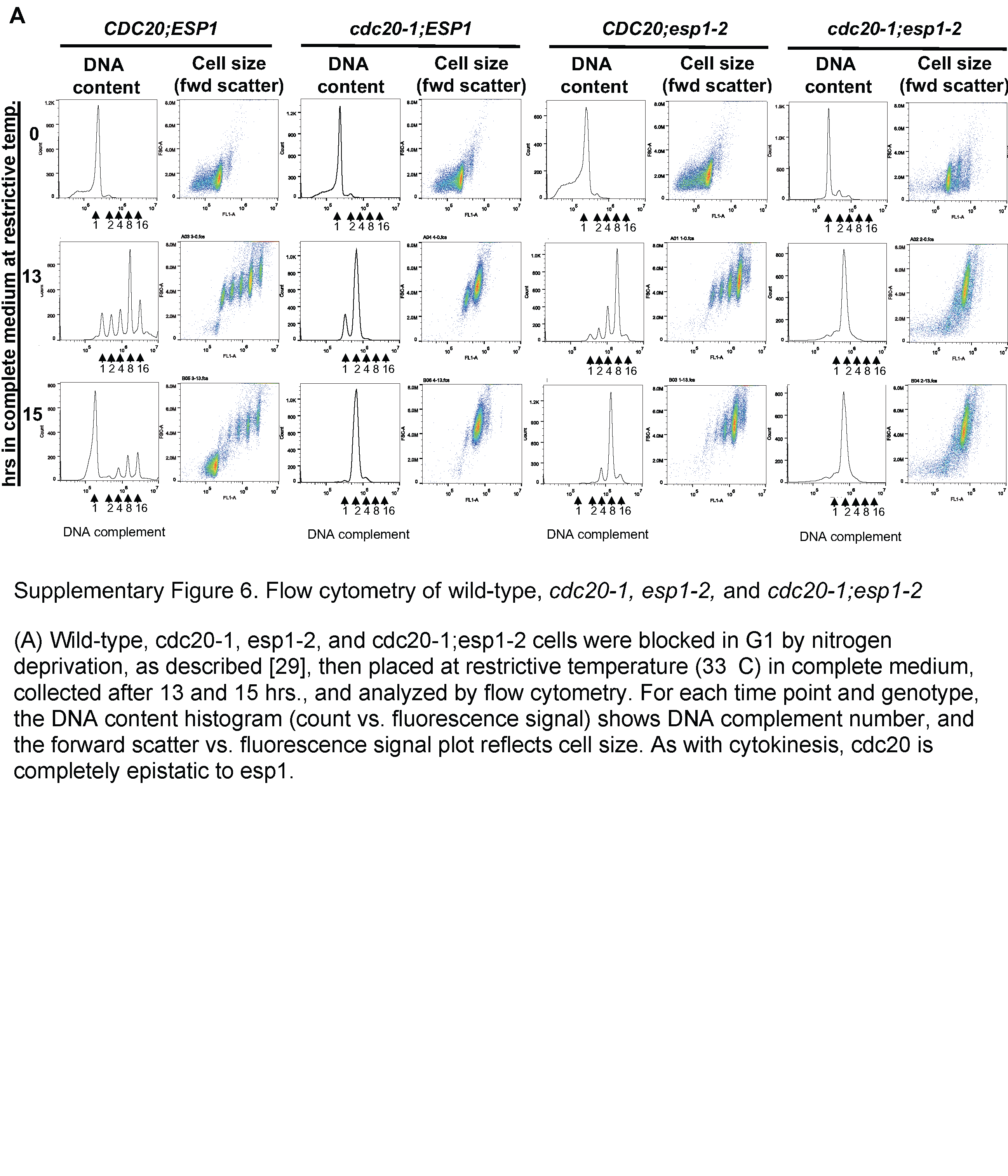

Supplement: S6 Fig — (A) Wild-type, cdc20-1, esp1-2, and cdc20-1;esp1-2 cells were blocked in G1 by nitrogen deprivation, as described [29], then placed at restrictive temperature (33 C) in complete medium, collected after 13 and 15 hrs., and analyzed by flow cytometry. For each time point and genotype, the DNA content histogram (count vs. fluorescence signal) shows DNA complement number, and the forward scatter vs. fluorescence signal plot reflects cell size. As with cytokinesis, cdc20 is completely epistatic to esp1. (TIFF) [file pgen.1009997.s006.tiff]

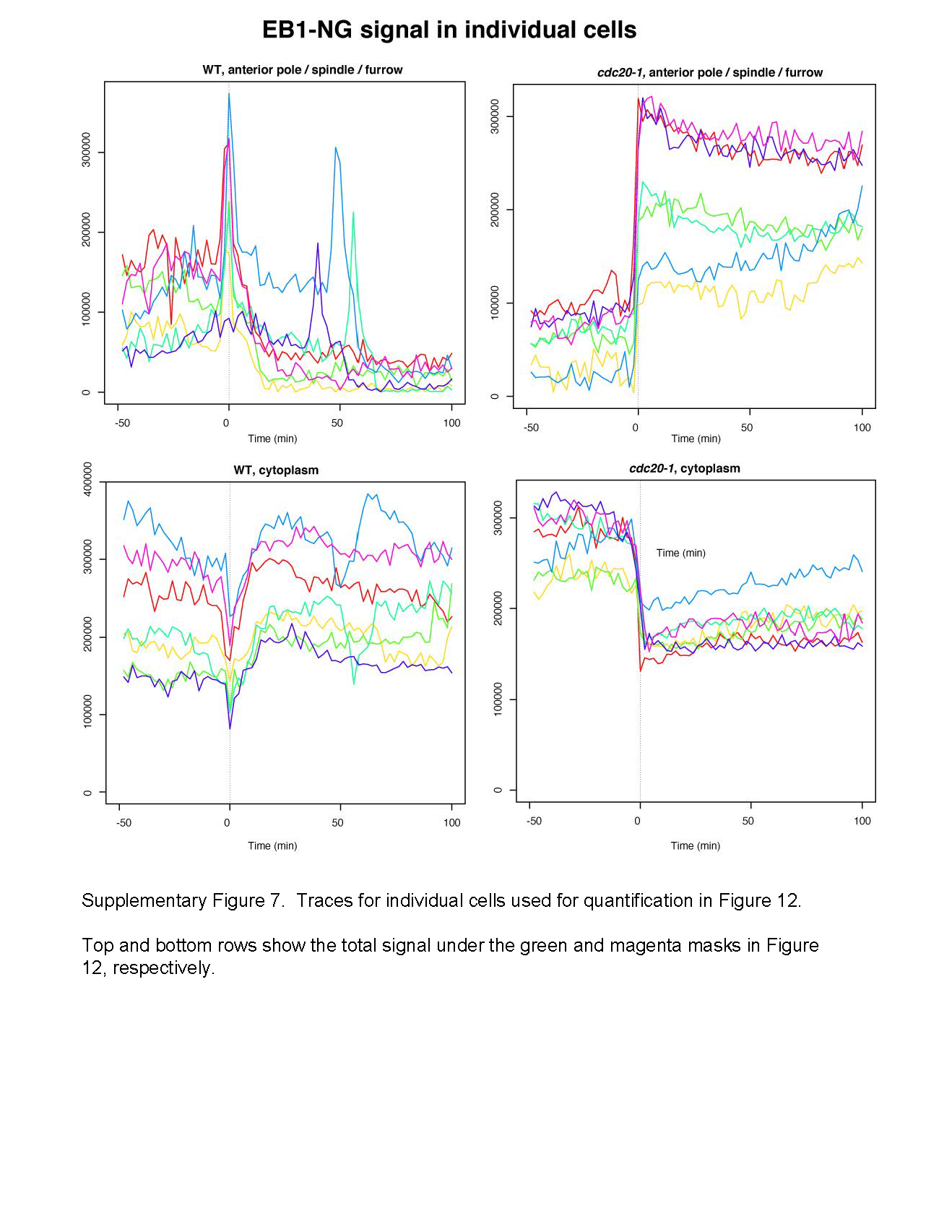

Supplement: S7 Fig — Top and bottom rows show the total signal under the green and magenta masks in Fig 12, respectively. (TIFF) [file pgen.1009997.s007.tiff]
